# Comparative efficacy and hematologic safety of different dosages of JAK inhibitors in the treatment of myelofibrosis: a network meta-analysis

**DOI:** 10.3389/fonc.2024.1403967

**Published:** 2024-08-30

**Authors:** Ke Chen, Yanyu Zhang, Jixuan Zou, Dehao Wang, Xinyue Yu, Yan Sun, Yumeng Li, Jicong Niu, Yi Chen, Pei Zhao, Weiyi Liu, Yan Lv, Mingjing Wang, Xiaomei Hu

**Affiliations:** ^1^ Postdoctoral Research Station of China Academy of Chinese Medical Sciences, Beijing, China; ^2^ Department of Hematology, Xiyuan Hospital, China Academy of Chinese Medical Sciences, Beijing, China; ^3^ Graduate School, China Academy of Chinese Medical Sciences, Beijing, China; ^4^ Graduate School, Beijing University of Chinese Medicine, Beijing, China

**Keywords:** myelofibrosis, JAK inhibitor, different doses, hematological safety, network meta-analysis

## Abstract

**Background:**

Myelofibrosis (MF) is a myeloproliferative neoplasm characterized by bone marrow fibrosis associated with substantial morbidity and mortality. The therapeutic landscape for MF has advanced with the development of Janus kinase inhibitors (JAKis) like ruxolitinib (RUX), fedratinib (FED), pacritinib (PAC), and momelotinib (MMB), aiming to alleviate symptoms and enhance patient comfort.

**Methods:**

A network meta-analysis was conducted to assess the efficacy and safety of eleven JAKi treatment regimens across nine randomized controlled trials (RCTs) with a total of 2340 participants. Outcomes were evaluated in terms of spleen volume reduction (SVR), total symptom score reduction (TSSR), hematological safety profiles, and overall survival (OS).

**Results:**

RUX and MMB were superior in achieving SVR and TSSR, with significant dose-response relationships observed. PAC and MMB were associated with a decreased risk of grade 3/4 anemia and thrombocytopenia compared to other JAKis. However, no substantial benefits in OS were observed with newer JAKis compared to RUX. The poorer OS outcomes with certain PAC dosages were likely influenced by baseline patient characteristics, particularly severe cytopenias.

**Conclusion:**

The introduction of JAKis significantly changed the treatment of MF. This meta-analysis reaffirms the core role of RUX and positions MMB as a potentially powerful alternative for treating symptoms and reducing spleen size. Meanwhile, MMB and PAC have a positive effect on anemia in MF while FED is more tolerable for patients with thrombocytopenia. However, it should be noted that these results are influenced by baseline patient characteristics, particularly cytopenias, which affects both management and overall survival. Therefore, there is an urgent need for personalized dosing strategies to optimize the balance between efficacy and safety, with careful consideration of patient-specific factors.

**Systematic review registration:**

https://www.crd.york.ac.uk/PROSPERO/, identifier CRD42023424179.

## 1 Introduction

Myelofibrosis (MF) is chronic myeloproliferative neoplasms (MPNs) associated with bone marrow fibrosis. It may cause bone marrow failure and has the potential to progress to acute myeloid leukemia (AML), primarily due to abnormal cytokine expression and transformation into accelerated and blast phases (1). Primary MF (PMF) has an incidence rate of approximately 0.3 to 0.6 cases per 100,000 individuals, typically affecting individuals aged 69 years, with a median overall survival (OS) of 3.6 years (2, 3). Unfortunately, over 80% of patients are at an intermediate or high risk of MF, resulting in shorter overall survival. Leading causes of death among MF patients include heart failure, vascular complications, and AML (2). MF is classified into PMF or secondary MF (SMF), occurring alongside other MPNs, such as post-polycythemia vera myelofibrosis (PPV-MF) or post-essential thrombocythemia myelofibrosis (PET-MF) (4). Progression of MF is associated with increasing splenomegaly, leading to symptoms like abdominal distension, pain, dyspnea, and splenic infarction, alongside worsening constitutional symptoms and cytopenias (5).

Advancements in molecular testing have identified driver mutations in the Janus kinase 2 (JAK2) gene in approximately 60% of MF patients (6). This discovery led to the development of JAK inhibitors (JAKis) like ruxolitinib (RUX), fedratinib (FED), pacritinib (PAC), and momelotinib (MMB), as therapeutic interventions for PMF and SMF. RUX, FED, and PAC have gained Food and Drug Administration (FDA) approval due to their positive impact on constitutional symptoms and splenomegaly through effective inhibition of inflammatory cytokines and myeloproliferation, significantly improving the quality of life for MF patients.

A previous systematic review focused on the effectiveness and tolerability of JAK inhibitors, establishing RUX as the primary JAKi for reducing spleen enlargement and improving disease-related symptoms, closely followed by FED (7). Besides, MMB displayed promising efficacy and safety in treating MF; however, evidence was limited due to the scarcity of included studies. However, differences between various JAK inhibitors and their combinations were not assessed. With the publication of several clinical studies (8–10), a comprehensive assessment of combined JAKi doses has become feasible. Based on previous research, we have incorporated data from two additional RCTs and differentiated among various dosages of JAK inhibitors. Also, both direct and indirect evidence has been synthesized using a network meta-analysis and systematic review. Therefore, this approach aids in comparing the efficacy and safety of different JAK inhibitors and dosages, ultimately furnishing valuable evidence for clinical decision-making in MF treatment.

## 2 Methods

### 2.1 Protocol registration

Before initiating the systematic review, this study was pre-registered in the PROSPERO database (CRD42023424179) (11).

### 2.2 Literature search

A comprehensive literature search was conducted independently by two researchers for relevant randomized controlled trials (RCTs) published up to March 31, 2023 using PubMed, EMBASE, and the Cochrane Library databases. Search terms primarily include “myelofibrosis” and “randomized controlled trial”. Additionally, the bibliographies of retrieved articles were further scrutinized to ensure a thorough search. The detailed search strategy is delineated in **Supplementary Materials 1**.

### 2.3 Study selection

Eligible articles screened by two reviewers (Ke Chen and Yanyu Zhang) had to meet the following predefined inclusion criteria: (1) Study involving patients diagnosed with MF, including essential PMF, PPV-MF, and PET-MF. (2) Intervention measures include treatment with JAK inhibitors. (3) Outcome measurements include spleen volume reduction (SVR) of over 35% and total symptom score reduction (TSSR) of over 50% post 24 weeks of treatment. Notably, significant adverse events, specifically severe thrombocytopenia (grade 3 or 4) and severe anemia (grade 3 or 4), were monitored during this period. Severe anemia and thrombocytopenia were defined as hemoglobin levels below 8 g/dL and platelet counts below 50 x 10^9/L, respectively. (4) Study designs include only RCTs.

### 2.4 Data extraction

Data extraction was meticulously performed by two reviewers (Ke Chen and Yanyu Zhang), encompassing details like publication year, study design, patient demographics (number, age, sex), intervention specifics, comparison details, duration of treatment and follow-up, along with recorded outcomes. All data observed during both the treatment and follow-up periods were systematically documented.

### 2.5 Quality assessment

The quality assessment and risk of bias were evaluated by two reviewers (Ke Chen and Yanyu Zhang), and the included RCTs were assessed following the Cochrane Handbook for Systematic Reviews of Interventions (12), Evaluated domains included randomization, allocation concealment, blinding, data completeness, selective reporting, and other potential biases. Each study was subsequently assigned a risk of bias rated with low, unclear, or high. Results were recorded in a comprehensive risk of bias table.

### 2.6 Statistical analysis

Data presentation involved tabulating event counts, sample sizes, study characteristics, and patient demographics, categorized by specific drug treatments. Forest plots graphically represented incidences of specific events or outcomes for each treatment arm or study, accompanied by binomial 95% confidence intervals (CI). Comparative trials provided risk ratio estimates. Study heterogeneity was quantified using the *I^2^* statistic, with an *I^2^* > 50% indicating substantial heterogeneity. In cases where heterogeneity was detected, sensitivity analyses were performed to identify the source of heterogeneity.

All statistical analyses were conducted using R software version 4.2.2 (13). To ensure the methodological robustness of the network meta-analysis, the PSRF (Potential Scale Reduction Factor) values were calculated for our analytical outcomes, with a value of 1 indicating satisfactory convergence. Furthermore, the mtc.nodesplit and mtc.anohe functions from the gemtc package were used to scrutinize the assumptions of consistency and homogeneity in our network meta-analysis, respectively. A p≥0.05 suggested consistency, and I^2^≤50% was indicative of homogeneity. Network meta-analysis was visualized through network plots, excluding trials without interventional connections. Markov chain Monte Carlo (MCMC) methods summarized posterior distributions (14), employing non-informative uniform and normal prior distributions, with the MCMC algorithm initiating from a random starting point (15). Treatment rankings for each outcome were determined using median rank (MR) and surface under the cumulative ranking area (SUCRA) calculations (16).

To enhance the robustness and reliability of this study, CINeMA (Confidence in Network Meta-Analysis) was adopted to conduct a reliability assessment for all the results of this study. It is a standardized evaluation method for assessing the credibility of network meta-analysis results. This evaluation comprehensively assesses the reliability of the evidence by considering factors such as the risk of bias, indirectness, imprecision, heterogeneity, and publication bias of the studies. The certainty of evidence (COE) was finally used to assess the level of evidence of the results, which was classified into four levels: high, moderate, low, and very low (17).

## 3 Results

### 3.1 Study characteristics

**Figure 1:**
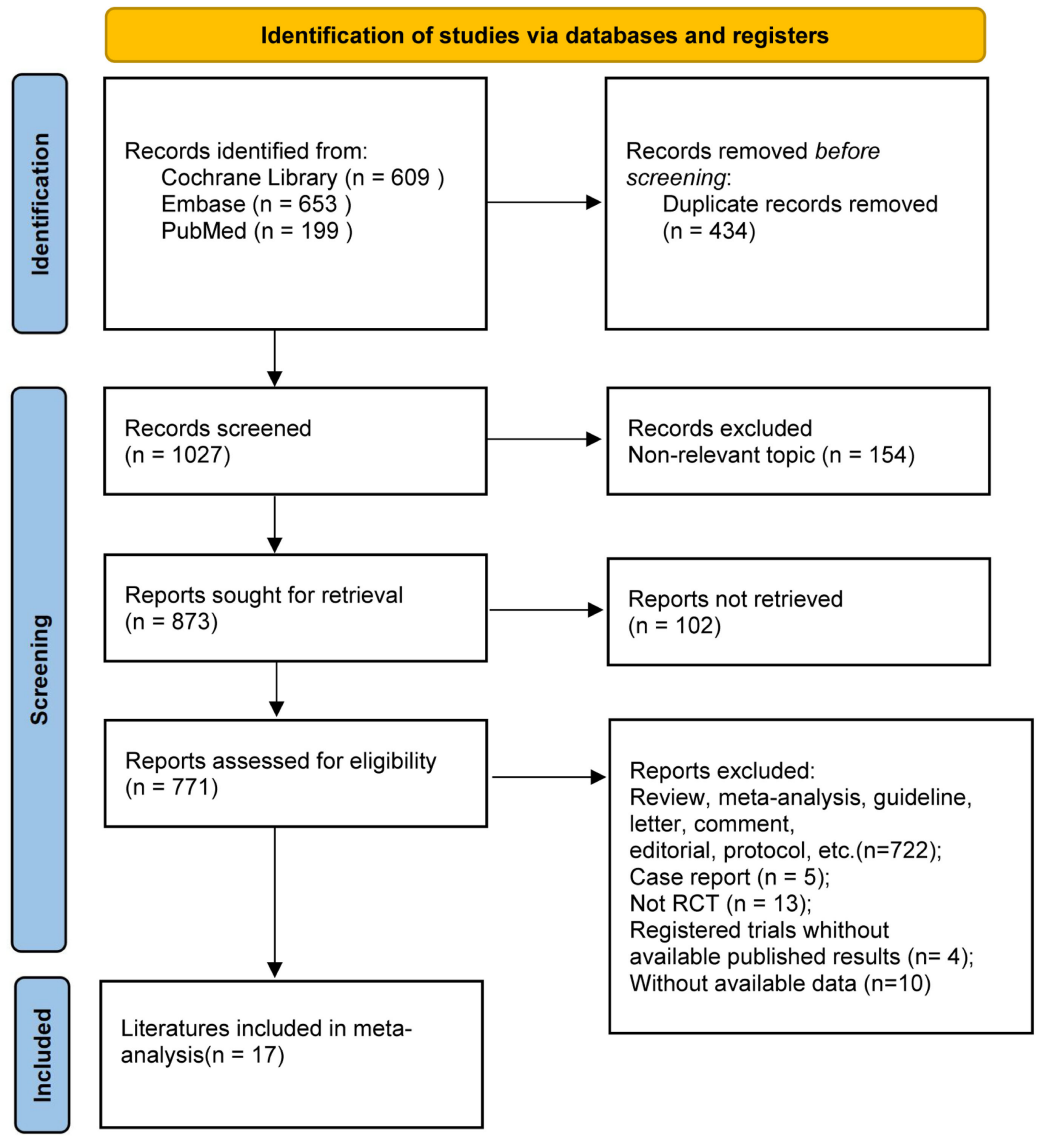
PRISMA flow diagram of literature screening.

An exhaustive search across multiple databases yielded a total of 1,461 articles. A total of 17 studies encompassing 9 RCTs were eligible and included in the meta-analysis (8–10, 18–31) (**Figure 1**). There were eleven treatment modalities: RUX, best available therapy (BAT), PLB (placebo), MMB 200 mg QD, PAC 400 mg QD, PAC 200 mg bid, PAC 100 mg QD, PAC 100 mg bid, FED 300 mg QD, FED 400mg QD, and FED 500 mg QD.

The included studies enrolled a total of 2,340 adult patients diagnosed with PPV-MF (25.0%), PMF (58.7%), or PET-MF (16.2%). Due to the combined presence of primary and secondary myelofibrosis across all studies, a subgroup analysis based on etiology was not feasible. Three of the included trials were multinational studies, recruiting patients with MF to assess the efficacy of JAK inhibitors in comparison to BAT, PLB, or other JAK inhibitors. **Table 1** and **Supplementary Materials 2** provide a concise overview of the fundamental characteristics of these 17 studies. Notably, there were differences in baseline characteristics among the studies. Specifically, in the three studies of SIMPLIFY-2, PERSIST-2, and PAC203, their patients were previously exposed to JAKi, while in other studies, the included patients had no previous JAKi exposure. In terms of the evaluation of TSSR efficacy, MF-TSS evaluation method was adopted in the COMFORT-I and JAKARTA studies, while the MPN-TSS evaluation method was used in the SIMPLIFY-1, SIMPLIFY-2, and PERSIST-1 studies. Among the included study populations, the platelet and hemoglobin levels of some patients were lower than normal in some studies. Among them, the platelet counts of patients included in the PAC203, PERSIST-1, PERSIST-2, and SAR302503 studies were lower than normal. The hemoglobin levels of patients included in the PAC203, PERSIST-1, PERSIST-2, and SIMPLIFY-2 studies were lower than 10 grams per deciliter. The above-mentioned differences may have a certain impact on the reliability of the results. Subgroup analyses and CINeMA evaluations were further conducted to objectively evaluate and present the reliability of the study results (**Supplementary Materials**).

**Table 1:** Characteristics of included trials.

First Author	Trial name, yr	Region	Study Population	Treatment arms	Previous JAKi exposure	Minimal treatment period
Verstovsek, S.	COMFORT-I, 2012	USA	309 patients mean age: 67.74, 9.01 Primary/Secondary MF: 154, 155	RUX vs. PLB	No	24 weeks
	COMFORT-I, 2013					
	COMFORT-I, 2015					
	COMFORT-I, 2017					
Harrison, C.	COMFORT-II, 2012	USA	219 patients mean age: 65.92, 9.59 Primary/Secondary MF: 116, 102	RUX vs. BAT	No	24 weeks
Cervantes, F.	COMFORT-II, 2013					
Mesa, R.	COMFORT-II, 2013					
Harrison, CN.	COMFORT-II, 2016					
Pardanani, A.	JAKARTA, 2015	Multinational	289 patients mean age: 64.05, 9.89 Primary/Secondary MF: 183, 106	FED 400mg qd FED 500mg qd vs. PLB	No	24 weeks
	JAKARTA, 2021					
Gerds, AT.	PAC203, 2015	USA	161 patients mean age: 68.94, 2.12 Primary/Secondary MF: 93, 68	PAC 100mg qd PAC 100mg bid PAC 200mg bid	Yes, after RUX exposure	24 weeks
Mesa, R.	PERSIST-1, 2017	Multinational	327 patients mean age: 66.97, 2.35 Primary/Secondary MF: 203, 124	PAC 400mg qd vs. BAT	No	24 weeks
Mascarenhas, J.	PERSIST-2, 2018	USA	221 patients mean age: 67.26, 10.71 Primary/Secondary MF: 144, 77	PAC 400mg qd PAC 200mg bid vs. BAT	Previous RUX exposure or not	24 weeks
Pardanani, A.	SAR302503, 2020	USA	31 patients mean age: 64.56, 11.83 Primary/Secondary MF: 18, 13	FED 300mg qd FED 400mg qd FED 500mg qd	No	24 weeks
Mesa, R.	SIMPLIFY-1, 2017	USA	432 patients mean age: 64.70, 10.62 Primary/Secondary MF: 244, 188	MMB 200mg qd vs. RUX 20mg bid	No	24 weeks
	SIMPLIFY-1, 2022					
Harrison, CN.	SIMPLIFY-2, 2018	Multinational	156 patients mean age: 67.4, 7.98 Primary/Secondary MF: 94, 62	MMB 200mg qd vs. BAT (include rux)	Yes, after RUX exposure	24 weeks

MF, myelofibrosis; RUX, ruxolitinib; FED, fedratinib; PAC, pacritinib; MMB, momelotinib; BAT, best available therapy; qd, once daily; bid, twice a day.

### 3.2 Risk of bias

Studies included in this meta-analysis were all RCTs. Among these, three trials were meticulously designed as double-blind studies. The trials, namely JAKARTA, SAR302503, and SIMPLIFY-2, were open-label studies. The quality of RCTs was acceptable.

**Figure 2:**
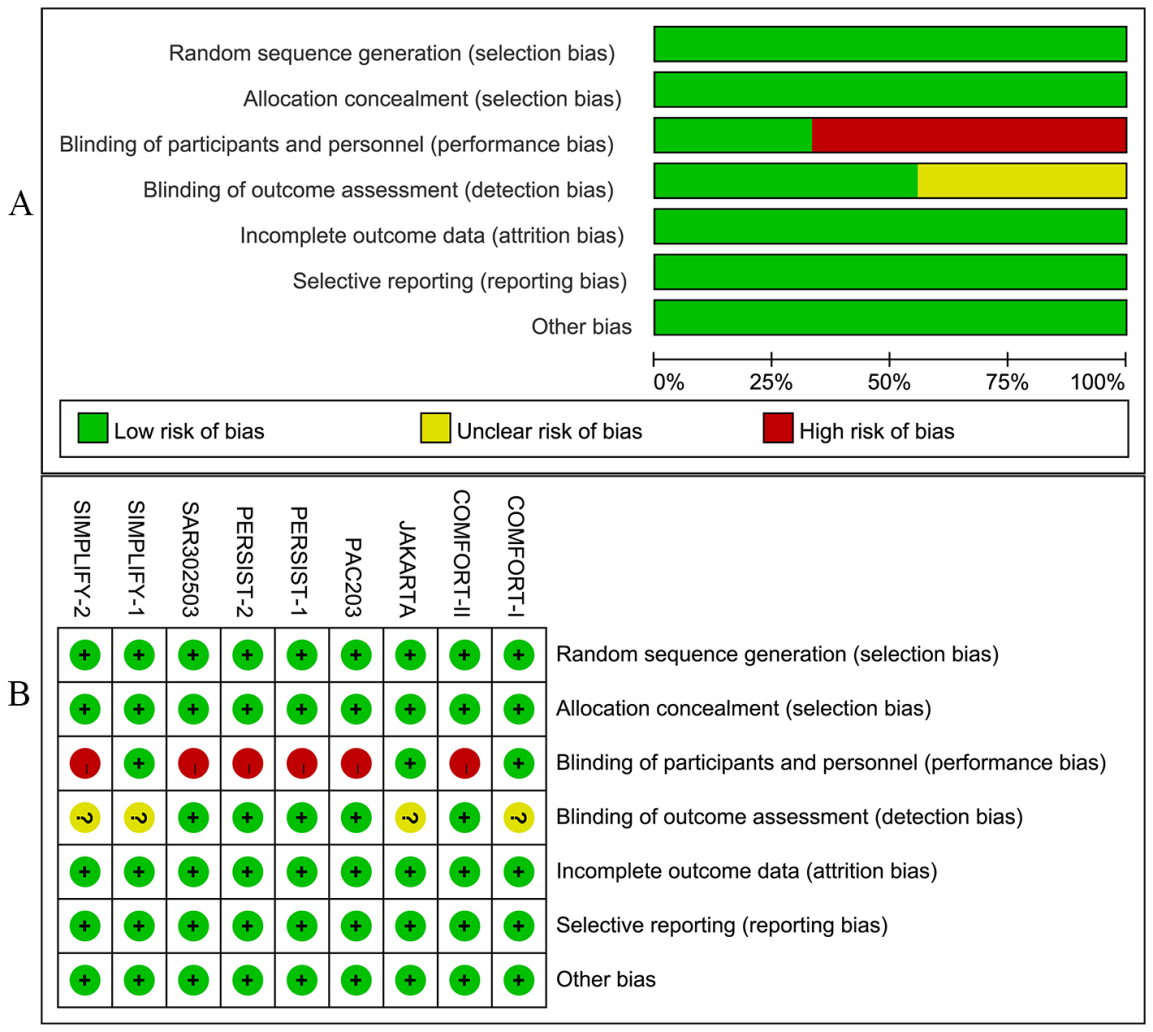
**(A)** The risk of bias graph; **(B)** The risk of bias summary.

In addition, the included studies provided detailed information on patient demographics, disease profiles, and inclusion criteria (**Supplementary Materials 3**, **4**). **Figures 2A, B** illustrate the bias graph and the summary of bias risk, respectively.

### 3.3 Network of treatment efficacy and safety

**Figure 3:**
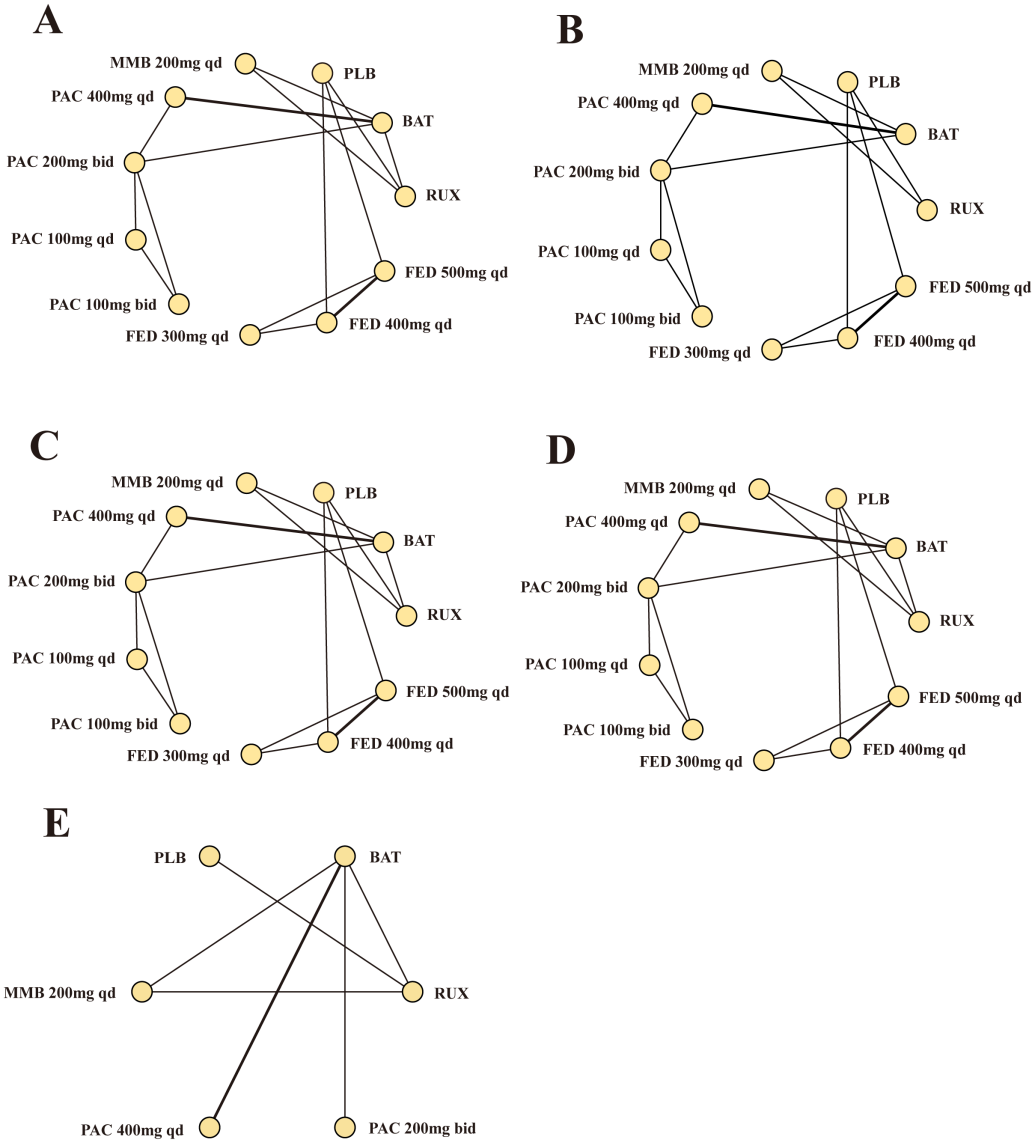
Network meta-analysis comparing different JAK inhibitors for MF patients. **(A)** Comparison of SVR. **(B)** Comparison of TSSR. **(C)** Comparison of grade 3/4 anemia. **(D)** Comparison of grade 3/4 thrombocytopenia. **(E)** Comparison of overall survival. MF, myelofibrosis; RUX, ruxolitinib; FED, fedratinib; PAC, pacritinib; MMB, momelotinib; BAT, best available therapy; QD, once daily; bid, twice a day.

Our network meta-analysis synthesized data from nine RCTs to compare the efficacy and safety across eleven treatment modalities (**Figure 3**). These modalities included MMB, PAC at various doses, FED, RUX, BAT, and PLB. The densely interconnective networks indicated a robust comparison of multiple treatment arms, which facilitated the evaluation of the efficacy of the studied JAK inhibitors for SVR.

### 3.4 Efficacy outcomes

All network meta-analysis analyses in the present study yielded PSRF values equal to 1, indicating overall satisfactory convergence. Additionally, both the consistency and homogeneity assumptions were met across all analyses. Details regarding the tests of the consistency and homogeneity are provided in **Supplementary Materials**.

#### 3.4.1 SVR

In our network meta-analysis of JAK inhibitors for MF, significant differences in treatment efficacy were observed. The quantified RRs with corresponding 95% CrIs revealed notable differences in the safety profiles of these inhibitors. Additionally, according to the SUCRA values, the relative safety of the different JAK inhibitors was ranked.

**Figure 4:**
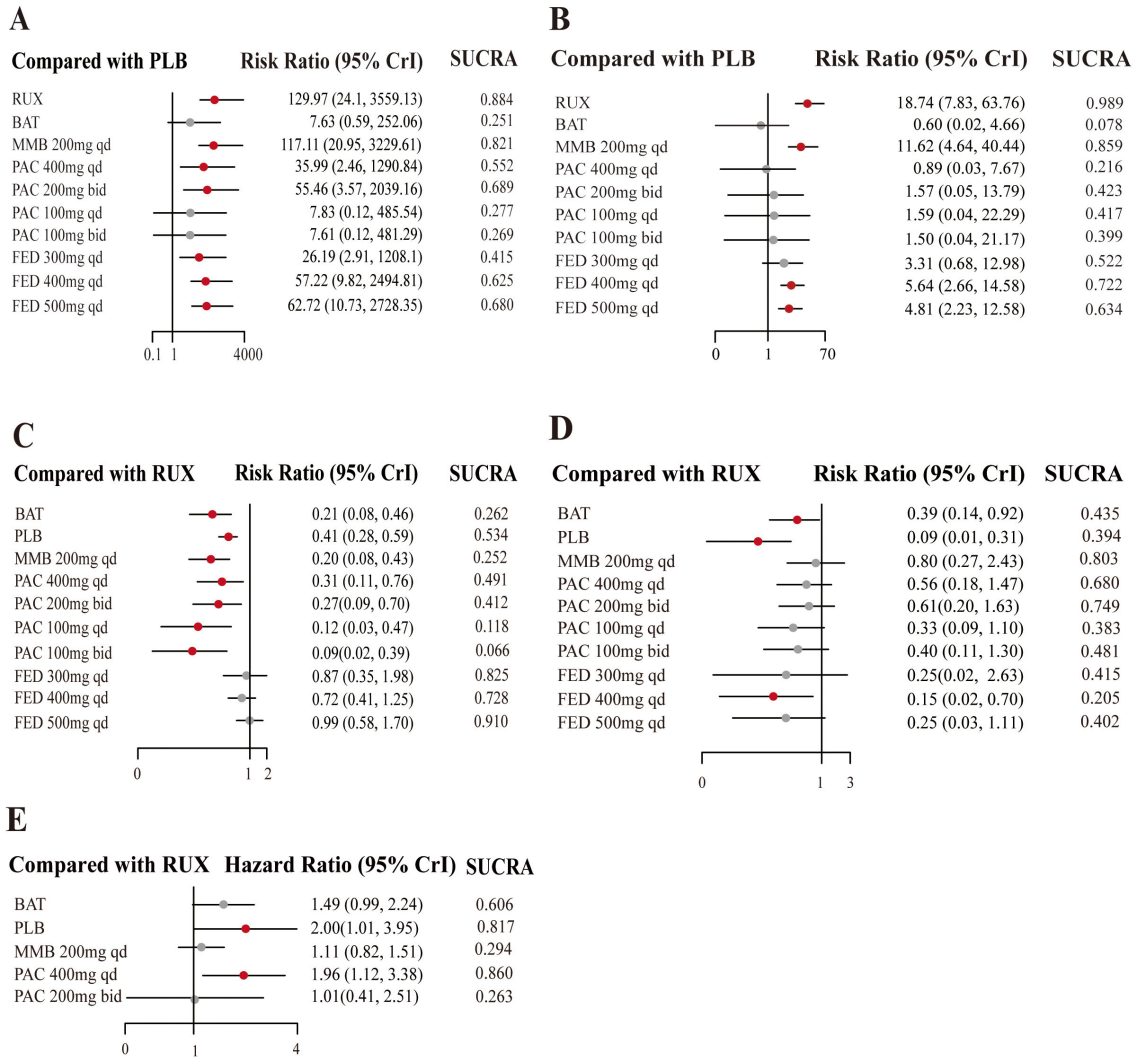
Forest plots of JAKi efficacy. Estimates of risk in the intention-to-treat population for **(A)** SVR, in the per protocol population for **(B)** TSSR, in the intention-to-treat population for **(C)** grade 3/4 anemia events, and for **(D)** grade 3/4 thrombocytopenia events. The meta-analysis of overall survival **(E)**.

RUX emerged as the most efficacious option for SVR (RR = 129.97, 95% CrI: 24.1-3559.13, CINeMA Moderate COE) (**Figure 4A**) and had the highest probability of being the most effective treatment for MF (SUCRA = 0.884) (**Table 2**). Similarly, the efficacy of MMB at a dosage of 200 mg daily was significant (RR = 117.11, 95% CrI: 20.95-3229.61, CINeMA Moderate COE) and MMB could serve as a robust alternative treatment option (SUCRA = 0.821).

**Table 2:** Comparison of the efficacy of JAkis.

**RUX**	**●0.03** **(0, 0.17)**	**●0.05** **(0.02, 0.13)**	**●0.62** **(0.48, 0.8)**	**●0.05** **(0, 0.28)**	**●0.08** **(0, 0.5)**	**●0.08** **(0, 0.89)**	**●0.08** **(0, 0.84)**	**●0.17** **(0.02, 0.9)**	**●**0.3 (0.07, 1.09)	**●0.26** **(0.06, 0.94)**
**●16.8** **(5.04, 112.75)**	**BAT**	**●**1.67 (0.21, 48.98)	**●19.37** **(3.83, 519.78)**	**●**1.49 (0.85, 2.75)	**●2.62** **(1.43, 5.09)**	**●**2.75 (0.57, 13.49)	**●**2.61 (0.54, 12.81)	**●**5.59 (0.42, 198.15)	**●9.79** **(1.04, 308.93)**	**●**8.34 (0.88, 264.03)
**●129.48** **(24.46, 3374.21)**	**●**7.69 (0.6, 236.93)	**PLB**	**●11.62** **(4.64, 40.44)**	**●**0.89 (0.03, 7.67)	**●**1.57 (0.05, 13.79)	**●**1.59 (0.04, 22.29)	**●**1.5 (0.04, 21.17)	**●**3.31 (0.68, 12.98)	**●5.64** **(2.66, 14.58)**	**●4.81** **(2.23, 12.58)**
**●**1.11 (0.82, 1.51)	**●0.07** **(0.01, 0.23)**	**●0.01** **(0, 0.05)**	**MMB 200mg qd**	**●0.08** **(0, 0.44)**	**●0.13** **(0, 0.79)**	**●**0.13 (0, 1.41)	**●**0.13 (0, 1.34)	**●**0.27 (0.04, 1.48)	**●**0.49 (0.11, 1.82)	**●**0.41 (0.1, 1.56)
**●**3.68 (0.81, 27.58)	**●0.22** **(0.09, 0.44)**	**●0.03** **(0, 0.39)**	**●**3.31 (0.71, 25.13)	**PAC 400mg qd**	**●1.76** **(1.02, 3.07)**	**●**1.84 (0.39, 8.74)	**●**1.75 (0.37, 8.28)	**●**3.75 (0.26, 139.3)	**●**6.57 (0.64, 213.7)	**●**5.6 (0.54, 182.67)
**●**2.42 (0.48, 19.3)	**●0.14** **(0.05, 0.35)**	**●0.02** **(0, 0.27)**	**●**2.18 (0.43, 17.6)	**●**0.66 (0.32, 1.3)	**PAC 200mg bid**	**●**1.04 (0.24, 4.52)	**●**0.99 (0.23, 4.28)	**●**2.12 (0.14, 79.73)	**●**3.72 (0.36, 120.44)	**●**3.17 (0.3, 103.59)
**●17.51** **(1.19, 787.09)**	**●**0.94 (0.1, 29.44)	**●**0.12 (0, 8.37)	**●15.77** **(1.05, 709.12)**	**●**4.36 (0.53, 131.81)	**●**6.53 (0.93, 190.59)	**PAC 100mg qd**	**●**0.95 (0.22, 4.01)	**●**2.06 (0.1, 99.34)	**●**3.67 (0.23, 155.12)	**●**3.13 (0.19, 131.26)
**●18.1** **(1.24, 811.17)**	**●**0.97 (0.1, 29.76)	**●**0.13 (0, 8.51)	**●16.23** **(1.1, 730.76)**	**●**4.51 (0.55, 132.05)	**●**6.79 (0.96, 190.23)	**●**1.04 (0.03, 38.27)	**PAC 100mg bid**	**●**2.18 (0.1, 104.37)	**●**3.86 (0.24, 156.6)	**●**3.29 (0.2, 135.78)
**●**5.5 (0.09, 261.35)	**●**0.3 (0, 17.45)	**●0.04** **(0, 0.38)**	**●**4.95 (0.08, 235.67)	**●**1.42 (0.02, 88.19)	**●**2.16 (0.02, 139.93)	**●**0.29 (0, 32.53)	**●**0.28 (0, 31.92)	**FED 300mg qd**	**●**1.7 (0.62, 7.13)	**●**1.45 (0.54, 6.05)
**●**2.42 (0.04, 91.62)	**●**0.13 (0, 6.35)	**●0.02** **(0, 0.1)**	**●**2.17 (0.04, 84)	**●**0.63 (0.01, 32.09)	**●**0.95 (0.01, 50.99)	**●**0.13 (0, 12.1)	**●**0.12 (0, 11.82)	**●**0.46 (0.11, 1.14)	**FED 400mg qd**	**●**0.85 (0.59, 1.23)
**●**2.21 (0.04, 84.53)	**●**0.12 (0, 5.84)	**●0.02** **(0, 0.09)**	**●**1.99 (0.03, 76.72)	**●**0.57 (0.01, 29.63)	**●**0.87 (0.01, 46.81)	**●**0.12 (0, 11.15)	**●**0.11 (0, 10.81)	**●**0.43 (0.1, 1.03)	**●**0.92 (0.66, 1.26)	**FED 500mg qd**
			**SVR** **(RR with 95%IC)**		**TSSR** **(RR with 95%IC)**		**Treatment**			
							

**●**indicates that the result of the CINeMA evaluation method attains a high level of COE, and **●** indicates a Moderate level.

Additionally, the efficacy of treatment measures varied depending on the dosages, with particularly notable differences observed in PAC regimens. For instance, PAC at 200 mg administered twice daily was moderately effective (RR = 55.46, 95% CrI: 3.57-2039.16, CINeMA Moderate COE, SUCRA = 0.689). Moreover, higher SUCRA values indicated a greater probability of being the most effective measure. The SUCRA values for FED at daily dosages of 400 mg and 500 mg were 0.625 and 0.680, respectively.

To summarize, this analysis highlights a dose-response relationship in the efficacy of JAK inhibitors for SVR in MF patients, underscoring the potential benefits of personalized dosing strategies to improve patient outcomes. The SUCRA scores could be used to rank the effectiveness of treatments. RUX and MMB ranked top. These insights are critical for optimizing therapeutic regimens to enhance outcomes for patients.

#### 3.4.2 TSSR

This network meta-analysis assessed the effects of different dosages of JAK inhibitors on TSSR in MF treatment, compared to a placebo control. The results revealed that RUX was significantly effective in improving TSSR (RR = 18.74, 95% CrI: 7.83-63.76, CINeMA Moderate COE) (**Figure 4B**) and was most likely to be the most efficacious strategy (SUCRA = 0.989) (**Table 2**). MMB at a dosage of 200 mg daily also exhibited robust efficacy (RR = 11.62, 95% CrI: 4.64-40.44, CINeMA Moderate COE) and may be used as an effective alternative for symptom alleviation (SUCRA = 0.859). Additionally, FED at a dosage of 400 mg daily showed more favorable outcomes (RR = 5.64, 95% CrI: 2.66-14.58, CINeMA Moderate COE) and may lower symptom scores (SUCRA = 0.722). Also, PAC at different dosages showed varying effectiveness, and PAC at 200 mg twice daily yielded modest benefits (RR = 0.89; 95% CrI: 0.03-6.67, CINeMA Moderate COE). Overall, the efficacy of different dosages of JAK inhibitors varied in controlling MF symptoms. RUX was particularly effective.

### 3.5 Hematological safety

This network meta-analysis investigated the hematological safety of JAK inhibitors in the treatment of MF, particularly the risk of grade 3/4 anemia and thrombocytopenia across various treatment regimens in comparison to the standard treatment RUX.

The results highlighted PAC and MMB as relatively safer options, with g a lower risk of anemia. Specifically, MMB 200 mg daily was associated with the lowest risk (RR = 0.20, 95% CrI: 0.08-0.43, CINeMA High COE, SUCRA = 0.252) (**Figure 4C**). Conversely, FED 500 mg daily was associated with the highest risk of anemia (RR = 0.99, 95% CrI: 0.58-1.70, CINeMA High COE, SUCRA = 0.910) (**Table 3**).

**Table 3:** Comparison of the safety of Jakis.

**RUX**	**●0.21** **(0.08, 0.46)**	**●0.41** **(0.28, 0.59)**	**●0.2** **(0.08, 0.43)**	**●0.31** **(0.11, 0.76)**	**●0.27** **(0.09, 0.7)**	**●0.12** **(0.03, 0.47)**	**●0.09** **(0.02, 0.39)**	**●**0.87 (0.35, 1.98)	**●**0.72 (0.41, 1.25)	**●**0.99 (0.58, 1.7)
**●2.59** **(1.09, 7.31)**	**BAT**	**●**1.94 (0.82, 5.21)	**●**0.94 (0.46, 2.02)	**●**1.47 (0.99, 2.23)	**●**1.29 (0.76, 2.15)	**●**0.56 (0.16, 1.7)	**●**0.44 (0.11, 1.42)	**●4.08** **(1.21, 14.04)**	**●3.38** **(1.28, 9.92)**	**●4.68** **(1.8, 13.56)**
**●11.62** **(3.24, 81.28)**	**●**4.54 (0.85, 37.48)	**PLB**	**●**0.48 (0.19, 1.15)	**●**0.76 (0.26, 1.99)	**●**0.66 (0.22, 1.83)	**●**0.28 (0.06, 1.2)	**●0.22** **(0.04, 0.99)**	**●**2.1 (0.91, 4.41)	**●1.73** **(1.16, 2.65)**	**●2.39** **(1.66, 3.58)**
**●**1.24 (0.41, 3.72)	**●**0.48 (0.13, 1.42)	**●0.1** **(0.01, 0.58)**	**MMB 200mg qd**	**●**1.57 (0.67, 3.58)	**●**1.36 (0.54, 3.31)	**●**0.59 (0.14, 2.26)	**●**0.47 (0.1, 1.86)	**●4.36** **1.3, 14.22)**	**●3.59** **(1.38, 10.19)**	**●4.97** **(1.93, 13.91)**
**●**1.8 (0.68, 5.45)	**●**0.69 (0.46, 1.03)	**●0.15** **(0.02, 0.86)**	**●**1.44 (0.45, 5.42)	**PAC 400mg qd**	**●**0.87 (0.53, 1.4)	**●**0.38 (0.11, 1.12)	**●0.3** **(0.08, 0.94)**	**●**2.77 (0.76, 10.14)	**●**2.3 (0.79, 7.18)	**●3.18** **(1.12, 9.87)**
**●**1.64 (0.61, 5.04)	**●0.63** **(0.4, 0.98)**	**●0.14** **(0.02, 0.79)**	**●**1.32 (0.41, 5.05)	**●**0.91 (0.61, 1.36)	**PAC 200mg bid**	**●**0.44 (0.14, 1.15)	**●0.35** **(0.1, 0.96)**	**●**3.18 (0.85, 12.21)	**●**2.64 (0.87, 8.69)	**●3.65** **(1.22, 11.9)**
**●**3.03 (0.91, 11.46)	**●**1.15 (0.52, 2.72)	**●**0.26 (0.03, 1.67)	**●**2.42 (0.62, 11.1)	**●**1.66 (0.77, 3.86)	**●**1.82 (0.94, 3.84)	**PAC 100mg qd**	**●**0.79 (0.19, 2.98)	**●7.34** **(1.41, 41.68)**	**●6.1** **(1.36, 30.69)**	**●8.45** **(1.9, 42.34)**
**●**2.51 (0.77, 9.23)	**●**0.96 (0.45, 2.11)	**●**0.21 (0.02, 1.36)	**●**2.01 (0.53, 8.91)	**●**1.39 (0.66, 2.99)	**●**1.51 (0.82, 2.94)	**●**0.83 (0.38, 1.79)	**PAC 100mg bid**	**●9.35** **(1.69, 59.15)**	**●7.77** **(1.64, 43.46)**	**●10.76** **(2.3, 60.05)**
**●**3.94 (0.38, 63.77)	**●**1.51 (0.12, 27.36)	**●**0.32 (0.05, 2.62)	**●**3.2 (0.24, 61.59)	**●**2.18 (0.17, 41.19)	**●**2.38 (0.18, 44.8)	**●**1.3 (0.09, 26.71)	**●**1.56 (0.11, 31.72)	**FED 300mg qd**	**●**0.82 (0.43, 1.83)	**●**1.13 (0.61, 2.48)
**●6.53** **(1.42, 51.05)**	**●**2.52 (0.4, 23.51)	**●**0.56 (0.25, 1.17)	**●**5.35 (0.8, 53.73)	**●**3.65 (0.54, 35.95)	**●**4 (0.59, 39.28)	**●**2.18 (0.28, 23.37)	**●**2.63 (0.35, 27.71)	**●**1.73 (0.23, 10.23)	**FED 400mg qd**	**●1.38** **(1.06, 1.82)**
**●**3.97 (0.9, 30.8)	**●**1.54 (0.25, 14.14)	**●0.34** **(0.16, 0.66)**	**●**3.27 (0.5, 32.18)	**●**2.22 (0.34, 21.3)	**●**2.44 (0.37, 23.53)	**●**1.33 (0.18, 14)	**●**1.6 (0.22, 16.79)	**●**1.06 (0.14, 6.08)	**●**0.61 (0.35, 1.04)	**FED 500mg qd**
			**Thrombocytopenia** **≥grade 3** **(RR with 95%IC)**		**Anemia** **≥grade 3** **(RR with 95%IC)**		**Treatment**			
							
							

**●**indicates that the result of the CINeMA evaluation method attains a high level of COE.

Besides, PLB was associated with the lowest risk of thrombocytopenia (RR = 0.09, 95% CrI: 0.01-0.31, CINeMA High COE, SUCRA = 0.394) (**Figure 4D**, **Table 3**), followed by BAT (RR = 0.39, 95% CrI: 0.14-0.92, CINeMA High COE, SUCRA = 0.435). Additionally, FED 400 mg QD was associated with a reduced risk of thrombocytopenia (RR = 0.15, 95% CrI: 0.02-0.70, CINeMA High COE, SUCRA =0.205).

In summary, the study highlights the critical role of thoughtful selection of JAK inhibitor dosages in managing thrombocytopenia risk among MF patients The insights gained from the SUCRA values and RRs underscored the importance of personalized dosing strategies to achieve an optimal balance between therapeutic efficacy and hematological safety, particularly minimizing thrombocytopenia risk. However, the diversity of patient populations included in the study introduces variations, particularly in baseline platelet counts and hemoglobin levels. For instance, while many studies specified that peripheral blood blasts should be less than 10% and platelet counts should be at least 100 × 10^9^ per liter (as noted in the inclusion and exclusion criteria in **Table 3** of the **Supplementary Materials**), some studies, such as SIMPLIFY-2, did not clearly describe these conditions. Additionally, there were differences in the inclusion criteria for platelet counts among various studies, such as SAR302503 and PAC203, Approximately half of the incorporated studies failed to furnish the baseline values of PLT or HGB, and thereby it was arduous for us to undertake subgroup analyses (**Supplementary Materials 2**). These variations can significantly impact treatment management and OS outcomes, complicating the interpretation and generalizability of the results. It is crucial to consider these factors when evaluating the efficacy and safety of different JAK inhibitor regimens.

### 3.6 Overall survival

This network meta-analysis assessed the effects of JAK inhibitors on OS in the treatment of MF. Notably, PLB may adversely affect survival compared to active treatments (HR = 2.00, 95% CrI: 1.01-3.95, SUCRA = 0.817) (**Figure 4E**, **Table 4**). PAC 400 mg daily was also associated with a higher risk of poor OS (HR = 1.96, 95% CI: 1.12, 3.385, SUCRA =0.860). Compared to RUX, MMB 200 mg daily and PAC 200 mg twice daily did not demonstrate significant advantages.

**Table 4:** Comparison of overall survival.

**RUX**					
0.67 (0.45, 1.01)	**BAT**				
**0.5** **(0.25, 0.99)**	0.74 (0.34, 1.65)	**PLB**			
0.9 (0.66, 1.22)	1.35 (0.9, 2.01)	1.81 (0.85, 3.82)	**MMB 200mg qd**		
**0.51** **(0.3, 0.89)**	0.76 (0.53, 1.1)	1.03 (0.43, 2.46)	**0.57** **(0.33, 0.97)**	**PAC 400mg qd**	
0.99 (0.4, 2.45)	1.47 (0.65, 3.3)	1.97 (0.64, 6.11)	1.09 (0.44, 2.71)	1.93 (0.79, 4.71)	**PAC 200mg bid**
	**Overall survival** **(HR with 95%IC)**			**Treatment**	

In summary, these findings elucidate the diverse impacts of JAK inhibitor regimens on OS in patients with MF. It is noteworthy that compared to RUX, these newer agents do not exhibit significant advantages in terms of OS, and PAC at a daily dosage of 400 mg was inferior to RUX in terms of OS. Despite these findings, it is important to note that the poorer OS outcomes associated with specific PAC dosages may not solely reflect the efficacy of the treatment. The baseline characteristics of the patient populations, particularly those with severe cytopenias, play a significant role in OS outcomes. Patients with lower baseline platelet counts and hemoglobin levels tend to have poorer prognoses, which may have influenced the observed results.

## 4 Discussion

Our network meta-analysis, which integrates data from nine meticulously selected RCTs, ranks the efficacy and safety of JAK inhibitors in managing MF, thereby guiding clinical decisions in the treatment of MF.

Despite existing research in this area (7), the present study updates the existing knowledge by integrating data from two additional RCTs (9, 10). Moreover, we meticulously differentiate between various dosages of the same JAK inhibitor to provide direct comparisons among diverse types of JAK inhibitors and their respective dosages (27). This nuanced approach is essential for aiding clinicians in judiciously selecting JAK inhibitor dosages for individual patients (32). Our research corroborates the efficacy of MMB, which, provides a potential alternative to RUX, in contrast to previous studies (7). The comparative analysis elucidates that MMB is comparable to RUX in ameliorating splenomegaly and providing symptomatic relief, and it is more effective than FED. Notably, three studies, SIMPLIFY-2, PERSIST-2, and PAC203, included patients with previous JAKi exposure. Therefore, we conducted a subgroup analysis based on previous JAKi exposure. According to the results, among patients previously exposed to JAKi, none of the JAKi therapies showed superior efficacy and safety to MMB. Meanwhile, among patients not previously exposed to JAKi, RUX was more effective than MMB, but in terms of hematologic safety, MMB outperformed RUX. Additionally, other JAKi therapies were less effective than MMB. These findings are consistent with the results of the major analysis (**Supplementary Materials 4**).

Regarding FED, our findings delineate a pronounced dose-dependent relationship in its therapeutic efficacy, particularly in mitigating splenomegalia. In the JAKARTA study, the primary hematologic adverse event associated with FED was anemia. FED decreased hemoglobin levels, which reached the nadir between 12 and 16 weeks. Subsequently, mean hemoglobin levels were partially recovered in the FED 400 mg group, whereas such recovery was not observed in the FED 500 mg group. In the SAR30253 study, mean hemoglobin levels during treatment were lower in the FED 500 mg group than in other groups. Therefore, a starting dose of FED 400 mg once daily, approved by the FDA and EMA, is recommended, and the hemoglobin levels before FED treatment in patients should be of concern. It is recommended that patients be closely monitored for hemoglobin changes during treatment. Given both efficacy and safety, FED 400 mg daily emerges as the most favorable option, consistent with the conclusions of other studies (10, 33, 34). The SIMPLIFY-2, PERSIST-2, and PAC203 studies enrolled patients who had previously been treated with JAKi. To reduce the risk of bias, we conducted subgroup analyses. The results indicated that MMB was more effective and safer than FED and PAC. Notably, FED 500mg QD showed significantly poorer hematological safety than MMB. The trends observed in the subgroup analysis were generally consistent with our main findings (**Supplementary Materials 4**).

Furthermore, PAC is comparable to other JAK inhibitors in diminishing splenomegaly. However, PAC at a daily dose of 200 mg outperforms FED, different from previous reports. Despite the heterogeneity of assessment tools used to measure symptom improvement, subgroup analyses were conducted based on different assessment tools. Subgroup analysis indicated that FED 400 mg qd was comparable to RUX in relieving symptoms, followed by MMB. Conversely, PAC, BAT, and PLB were significantly inferior to RUX. These findings were generally in agreement with the results of the major analysis (**Supplementary Materials 5**).

Nonetheless, the use of JAK inhibitors may cause hematotoxicity, notably anemia and thrombocytopenia. These adverse effects are the predominant causes of intolerance and cessation of treatment among patients (35, 36). Our research suggests that an optimized dosage of JAK inhibitors may mitigate hematotoxicity, especially grade 3/4 anemia and thrombocytopenia.

Hematological safety evaluation revealed that the risk of grade 3/4 anemia in individuals receiving MMB and PAC was notably diminished in comparison to those receiving RUX and FED, different from earlier observations (7). Such findings provide a more accurate understanding of the pharmacodynamic properties of MMB and PAC and the hematotoxicity induced by varying dosages of JAK inhibitors, thereby assisting in the refinement of guidelines for their clinical utilization. Emerging evidence posits that reduced doses of MMB and PAC may serve as alternative therapeutic options for patients with MF who suffer from anemia without experiencing disabling symptomatic splenomegaly or systemic manifestations. These compounds are instrumental not only in alleviating symptoms and reducing splenomegaly but also in stimulating erythropoiesis. Despite the absence of approval from FDA for MMB, its efficacy in ameliorating anemia has been substantiated in phase II and III trials (37, 38). Further research has highlighted its beneficial impact on transfusion-dependent subjects (39). MMB and PAC, through the inhibition of Activin A receptor type I (ACVR1), enhance erythropoiesis and lessen the dependency on transfusions. The *in vitro* inhibitory concentrations of these agents are significantly lower than the therapeutic doses, thereby reducing adverse effects and presenting a viable strategy for the management of anemia (40). The PERSIST-1 clinical trial indicated a decrease by 25% in the demand for transfusion in patients on PAC (28).

Our meta-analysis also examined hematotoxicity, particularly grade 3/4 thrombocytopenia events. The hematotoxicity appeared to be less pronounced with FED, PAC, and MMB compared to RUX, deviating from previous research. Specifically, this study underscores that FED 400 mg daily significantly reduced hematotoxicity compared to RUX, whereas dosages of 300 mg daily and 500 mg daily failed to exhibit a definitive benefit. The JAKARTA and JAKARTA-2 trials specifically examined the safety and efficacy of FED at a daily dosage of 400 mg in individuals with platelet counts exceeding 50×10^9/L but not surpassing 100×10^9/L. Their results revealed that for patients falling within this platelet count range, FED 400 mg daily maintained similar safety and efficacy profiles (26, 27, 41). This further corroborates the diminished effect of FED on MF-related thrombocytopenia, consistent with the findings of our analysis. PAC has been approved for use in patients with platelet counts below 50×10^9/L. Emerging evidence posits that PAC, by inhibiting ACRV1, is instrumental in mitigating anemia (40). Within the PERSIST-1 trial, in comparison to BAT, PAC exhibited response rates of 19% versus 5% and 18% versus 3% (28). These findings not only validate the utilization of PAC in patients with platelet counts below 50×10^9/L but also support the use of JAK inhibitor therapy, especially when platelet counts fall below 100×10^9/L.

Since some studies (primarily involving PAC and FED) included patients with low platelet counts and hemoglobin levels below 10g/dL, there was a risk of bias. The PAC203, PERSIST-1, PERSIST-2, and SAR302503 studies included patients with low platelet counts, while the PAC203, PERSIST-1, PERSIST-2, and SIMPLIFY-2 studies included patients with hemoglobin levels below 10g/dL. Analysis indicated that in populations with low platelet counts and hemoglobin levels below 10g/dL, PAC 200mg Bid showed significantly superior efficacy and hematologic safety to BAT, consistent with previous research findings.

With unique pharmacological properties, newly developed JAK inhibitors confer diverse therapeutic effects in clinical practice. Specifically, MMB effectively reduces hepcidin expression, increases iron bioavailability for erythropoiesis, and significantly enhances the response to anemia (42). In contrast, PAC, compared to other JAK inhibitors, demonstrates reduced myelosuppression, offering a promising option for patients with MF and severe thrombocytopenia (43). Nevertheless, this study reveals that MMB, PAC, and BAT is not advantageous over RUX in improving survival. Furthermore, PAC 400 mg daily is associated with inferior OS in MF patients relative to RUX. Hence, despite potential limitations in the evidence base, it is imperative to exercise increased caution in selecting JAK inhibitors and their dosages.

Our investigation underscores the critical significance of tailored therapeutic approaches in MF management. Given the heterogeneity in baseline platelet counts observed across RCTs, we conducted rigorous sensitivity analyses (**Supplementary Materials 6**) and employed sophisticated statistical methodologies, including random-effects models. These techniques were instrumental in mitigating the influence of variability between studies, thereby enhancing the credibility of our findings concerning the relative efficacy and safety of JAK inhibitors in the context of MF.

Nevertheless, our study is subject to several constraints. Notably, the lack of available trials restricts the evidence base for our network meta-analysis, and the high diversity of study populations hampers our comprehensive assessment of publication bias. Furthermore, inconsistency tests are not performed, due to the absence of adequately closed loops. Furthermore, some meta-analysis results were derived from indirect comparisons. Such limitations may compromise the robustness and widespread relevance of our research outcomes. Future research should explore the differential efficacy of JAK inhibitors across different subgroups, including age, disease severity, or genetic mutations, to determine if certain populations derive greater benefits from specific treatments. Additionally, well-designed, large-scale, randomized, controlled, double-blind clinical studies are imperative to further evaluate the long-term safety and effectiveness of these therapies. Such studies may uncover novel therapeutic targets or combinations that could improve patient outcomes.The findings of this network meta-analysis highlight the heterogeneity in treatment efficacy and safety profiles of JAK inhibitors in MF patients. While the CINeMA framework has strengthened the certainty of evidence, it is critical to emphasize the role of baseline patient characteristics in interpreting these results. Patients with severe cytopenias often have poorer overall survival outcomes, and this is a significant confounding factor in evaluating treatment efficacy. Clinicians should be cautious in applying these results to practice, especially for patients with severe cytopenias. For these patients, treatment options may be limited, and PAC or MMB is a viable alternative despite their associated risks. Individualized treatment strategies that consider baseline patient characteristics and the severity of cytopenias are essential to optimize outcomes in real-world settings.

In conclusion, the introduction of JAK inhibitors has significantly changed the therapeutic landscape for myelofibrosis. This comprehensive meta-analysis reaffirms the efficacy of RUX and positions MMB as a potential alternative for symptom management and spleen size reduction. Meanwhile, MMB and PAC have a positive effect on anemia in MF while FED is more tolerable for patients with thrombocytopenia. However, it is imperative to interpret these findings with caution, considering the substantial influence of baseline cytopenias on overall survival and treatment outcomes. Personalized treatment strategies tailored to individual patient characteristics are necessary to maximize therapeutic benefits while minimizing risks. In this way, the findings can be applied appropriately in clinical practice, particularly for patients with severe cytopenias who have limited treatment alternatives.

## Data Availability

The original contributions presented in the study are included in the article/**Supplementary Material**. Further inquiries can be directed to the corresponding authors.

## Supplementary material

Supplementary Materials 1 Search strategy for study screening.

Supplementary Materials 2 Characteristics of included trials.

Supplementary Materials 3 Inclusion criteria.

Supplementary Materials 4 Exposed JAKi subgroup analysis.

Supplementary Materials 5 Total symptom score (TSS) Subgroup Analysis.

Supplementary Materials 6 Heterogeneity analysis.

Supplementary Materials 7 Consistency analysis.

Supplementary Materials 8 CINeMA confidence rating result.
