# Supplementary material for: Comparative efficacy and hematologic safety of different dosages of JAK inhibitors in the treatment of myelofibrosis: a network meta-analysis
Source: Front Oncol. 2024 Aug 30;14:1403967. doi: 10.3389/fonc.2024.1403967 (PMC11392783; doi:10.3389/fonc.2024.1403967)
Supplement: Supplementary Materials 1 — Search strategy for study screening. [file Table1.docx]

**Supplementary materials**

**1. Search strategy for study screening**

| **Database** | **Search strategy** | **Accessed on (date)** |
| --- | --- | --- |
| In Clinicaltrials.gov database | MeSH descriptor: Primary Myelofibrosis explode all trees OR ('Myelofibroses, Primary' OR 'Agnogenic Myeloid Metaplasia' OR 'Agnogenic Myeloid Metaplasias' OR 'Bone Marrow Fibroses' OR 'Bone Marrow Fibrosis' OR 'Chronic Idiopathic Myelofibrosis' OR 'Fibroses, Bone Marrow' OR 'Fibrosis, Bone Marrow' OR 'Idiopathic Myelofibrosis' OR 'Metaplasia, Agnogenic Myeloid' OR 'Metaplasia, Myeloid' OR 'Metaplasias, Agnogenic Myeloid' OR 'Metaplasias, Myeloid' OR 'Myelofibroses' OR 'Myelofibrosis' OR 'Myelofibrosis With Myeloid Metaplasia' OR 'Myelofibrosis, Primary' OR 'Myeloid Metaplasia' OR 'Myeloid Metaplasia,Agnogenic' OR 'Myeloid Metaplasias' OR 'Myeloid Metaplasias,Agnogenic' OR 'Myeloscleroses' OR 'Myelosclerosis' OR 'Myeloses, Nonleukemic' OR 'Myelosis, Nonleukemic' OR 'Nonleukemic Myeloses' OR 'Nonleukemic Myelosis' OR 'Primary Myelofibroses'):ti,ab,kw AND ('randomized controlled trial' OR 'Randomized' OR 'placebo'):ti,ab,kw | May 6, 2023 |
| In Embase database | 'primary myelofibrosis'/exp OR 'primary myelofibrosis':ti,ab,kw OR 'myelofibroses, primary':ti,ab,kw OR 'agnogenic myeloid metaplasia':ti,ab,kw OR 'agnogenic myeloid metaplasias':ti,ab,kw OR 'bone marrow fibroses':ti,ab,kw OR 'bone marrow fibrosis':ti,ab,kw OR 'chronic idiopathic myelofibrosis':ti,ab,kw OR 'fibroses, bone marrow':ti,ab,kw OR 'fibrosis, bone marrow':ti,ab,kw OR 'idiopathic myelofibrosis':ti,ab,kw OR 'metaplasia, agnogenic myeloid':ti,ab,kw OR 'metaplasia, myeloid':ti,ab,kw OR 'metaplasias, agnogenic myeloid':ti,ab,kw OR 'metaplasias, myeloid':ti,ab,kw OR 'myelofibroses':ti,ab,kw OR 'myelofibrosis':ti,ab,kw OR 'myelofibrosis with myeloid metaplasia':ti,ab,kw OR 'myelofibrosis, primary':ti,ab,kw OR 'myeloid metaplasia':ti,ab,kw OR 'myeloid metaplasia,agnogenic':ti,ab,kw OR 'myeloid metaplasias':ti,ab,kw OR 'myeloid metaplasias,agnogenic':ti,ab,kw OR 'myeloscleroses':ti,ab,kw OR 'myelosclerosis':ti,ab,kw OR 'myeloses, nonleukemic':ti,ab,kw OR 'myelosis, nonleukemic':ti,ab,kw OR 'nonleukemic myeloses':ti,ab,kw OR 'nonleukemic myelosis':ti,ab,kw OR 'primary myelofibroses':ti,ab,kw AND 'randomized controlled trial':ti,ab,kw OR 'randomized':ti,ab,kw OR 'placebo':ti,ab,kw | May 6, 2023 |
| In PubMed database | myelofibrosis AND "randomized controlled trial"Title/Abstract  Search details: "primary myelofibrosis"MeSH Terms OR "Agnogenic Myeloid Metaplasia"Title/Abstract OR "Bone Marrow Fibroses"Title/Abstract OR "Bone Marrow Fibrosis"Title/Abstract OR "Chronic Idiopathic Myelofibrosis"Title/Abstract OR "fibrosis bone marrow"Title/Abstract OR "Idiopathic Myelofibrosis"Title/Abstract OR "Myelofibroses"Title/Abstract OR "Myelofibrosis"Title/Abstract OR "Myelofibrosis With Myeloid Metaplasia"Title/Abstract OR "myelofibrosis primary"Title/Abstract OR "Myeloid Metaplasia"Title/Abstract OR "Myeloid Metaplasia,Agnogenic"Title/Abstract OR "Myeloid Metaplasias"Title/Abstract OR "Myeloscleroses"Title/Abstract OR "Myelosclerosis"Title/Abstract OR "Nonleukemic Myeloses"Title/Abstract OR "Nonleukemic Myelosis"Title/Abstract AND "randomized controlled trial"Title/Abstract OR "Randomized"Title/Abstract OR "placebo"Title/Abstract | May 6, 2023 |

**2.Characteristics of included trials.**

| **Study ID** | **Region** | **population** | **Age**  **（mean,SD）** | **Gender**  **(female,male)** | **MF subtype**  **(PMF/PPV/PET)** | **PLT** | **HGB** | **JAK2V617F Positive** | **IPSS RISK** | **Spleen size (cm）** | **spleen volume (cm3）** |
| --- | --- | --- | --- | --- | --- | --- | --- | --- | --- | --- | --- |
| COMFORT-I | USA | 309 | 67.74, 9.01 | 132, 167 | 154/97/57 | 272.04, 160.14 | 105.50, 23.03 | 191, 118 | High/Intermediate-2(189/118) | 16.16, 5.86 | 2728.69, 1451.42 |
| COMFORT-II | USA | 219 | 65.92, 9.59 | 94, 125 | 116/68/34 | 238.67,NA | NA | 136, 83 | High/Intermediate-2(131/87) | 14.81, 5.51 | 2563.85, 1411.54 |
| JAKARTA | Multinational | 289 | 64.05, 9.89 | 119, 170 | 183/76/30 | 253.07, 202.12 | 10.28, 2.50 | 193, 96 | High/Intermediate-2(139/150) | 16.27, 6.71 | 2723.63, 1429.68 |
| PAC203 | USA | 161 | 68.94, 2.12 | 69, 92 | 93/44/24 | 59.16, 15.96 | NA | NA | High/Intermediate-2/Intermediate-1(46/80/35) | 13.92, 2.38 | 2429.12, 436.18 |
| PERSIST-1 | Multinational | 327 | 66.97, 2.35 | 142, 185 | 203/81/42 | NA | NA | 246, 81 | High/Intermediate-2/Intermediate-1(47/106/173) | 12.0, 1.68 | 2066.38, 294.55 |
| PERSIST-2 | USA | 221 | 67.26, 10.71 | 96, 125 | 144/46/31 | NA | NA | 170, 51 | High/Intermediate-2/Intermediate-1(66/115/40) | 14.29, 6.24 | NA |
| SAR302503 | USA | 31 | 64.56, 11.83 | 15, 16 | 18/8/5 | NA | NA | 26, 5 | NA | NA | 2747.65, 1861.28 |
| SIMPLIFY-1 | USA | 432 | 64.70, 10.62 | 188, 244 | 244/98/90 | 301.30, 232.58 | 10.65, 2.24 | 266, 166 | High/Intermediate-2/Intermediate-1(200/143/89) | NA | NA |
| SIMPLIFY-2 | Multinational | 156 | 67.4, 7.98 | 63, 93 | 94/30/32 | 156.03, 134.24 | 9.43, 1.80 | 103, 53 | High/Intermediate-2/Intermediate-1(27/90/39) | NA | NA |

1. **Inclusion criteria**

| **Study ID** | **Inclusion Criteria** | **Exclusion Criteria** |
| --- | --- | --- |
| COMFORT-I | **Age:** 18 years or older.  **Diagnosis:** Primary myelofibrosis, post–polycythemia vera myelofibrosis, or post–essential thrombocythemia myelofibrosis according to 2008 World Health Organization criteria.  **Life Expectancy:** 6 months or longer.  **Risk Score:** An International Prognostic Scoring System (IPSS) score of 2 (intermediate-2 risk) or 3 or more (high risk).  **Performance Status:** An Eastern Cooperative Oncology Group performance status of 3 or less (on a scale from 0 to 5, with higher scores indicating greater disability).  **Peripheral-blood Blasts:** Less than 10%.  **Peripheral-blood CD34+ Cell Count:** More than 20×106 per liter.  **Platelet Count:** 100×109 per liter or more.  **Splenomegaly:** Palpable splenomegaly (≥5 cm below the left costal margin).  **Disease Status:** Disease that was refractory to available therapies, had side effects requiring their discontinuation, or were not candidates for available therapies and had disease requiring treatment. |  |
| COMFORT-II | **Age:** Patients must be 18 years of age or older.  **Diagnosis:** Patients must have primary myelofibrosis, post–polycythemia vera myelofibrosis, or post–essential thrombocythemia myelofibrosis.  **Palpable Spleen:** Patients must have a palpable spleen 5 cm or more below the costal margin.  **JAK2 V617F Mutation Status:** Patients are eligible irrespective of their JAK2 V617F mutation status.  **Prognostic Factors:** Patients must have either two prognostic factors (intermediate-2 risk) or three or more prognostic factors (high risk) according to the International Prognostic Scoring System.  **Peripheral-Blood Blast Count:** Patients must have a peripheral-blood blast count of less than 10%.  **Platelet Count:** Patients must have a platelet count of 100×10^9 or more per liter.  **Eastern Cooperative Oncology Group Performance Status:** Patients must have an Eastern Cooperative Oncology Group performance status of 3 or less.  **Prior Treatment with JAK Inhibitor:** Patients must not have received prior treatment with a JAK inhibitor.  **Suitability for Allogeneic Stem-Cell Transplantation:** Patients must not be considered suitable candidates for allogeneic stem-cell transplantation at the time of enrollment. |  |
| SIMPLIFY-1 | **Age:** Patients must be at least 18 years old.  **Palpable Splenomegaly:** Patients must have palpable splenomegaly of at least 5 cm below the left costal margin.  **Diagnosis:** Patients must have a confirmed diagnosis of primary myelofibrosis (according to WHO criteria) or post–polycythemia vera or post–essential thrombocythemia myelofibrosis (according to IWG-MRT criteria).  **Risk Classification:** Patients must be classified as International Prognostic Scoring System high risk, intermediate-2 risk, or intermediate-1 risk with symptomatic splenomegaly or hepatomegaly, or anemia (hemoglobin < 10.0 g/dL), and/or unresponsive to available non-JAKi therapy.  **Laboratory Tests:** Within 14 days before the first dose of study treatment, patients must meet the following laboratory test criteria:  Absolute neutrophil count ≥ 0.75 × 10^9/L (in the absence of growth factor therapy in the prior 7 days). Platelet count ≥ 50 × 10^9/L (≥ 100 × 10^9/L if AST or ALT ≥ 2 × ULN) (in the absence of platelet transfusion(s) or thrombopoietin mimetics in the prior 7 days). Peripheral blood blasts < 10%. AST and ALT ≤ 3 × ULN (≤ 5 × ULN if liver is involved by extramedullary hematopoiesis or related to iron chelator therapy started within the prior 60 days). Creatinine clearance ≥ 45 mL/min and direct bilirubin ≤ 2.0 × ULN.  **Eastern Cooperative Oncology Group Performance Status:** Patients must have an Eastern Cooperative Oncology Group performance status ≤ 2.  **Life Expectancy:** Patients must have a life expectancy > 24 weeks. | Prior use of a JAK inhibitor.  Prior splenectomy or spleen irradiation within 3 months before treatment initiation.  Certain cancers (history or concurrent disease).  Uncontrolled intercurrent illness limiting study compliance.  Eligible for allogeneic stem-cell transplantation. |
| SIMPLIFY-2 | **Age:** Patients must be at least 18 years old.  **Diagnosis:** Patients must have a confirmed diagnosis of primary myelofibrosis, polycythemia vera, or essential thrombocythemia myelofibrosis.  **Ruxolitinib Treatment:** Patients must have been currently or previously treated with ruxolitinib for at least 28 days and experienced either: Requirement for RBC transfusion on ruxolitinib, or Required a dose adjustment of ruxolitinib to less than 20 mg twice a day and also had: Anemia, Grade 3 thrombocytopenia, or Bleeding at grade 3 or worse.  **Palpable Spleen:** Patients must have a palpable spleen of at least 5 cm below the left costal margin.  **Peripheral Neuropathy:** Patients must not have grade 2 or greater peripheral neuropathy.  **DIPSS Classification:** Patients must be classified as DIPSS high risk, intermediate-2 risk, or intermediate-1 risk with symptomatic splenomegaly or hepatomegaly.  **ECOG Performance Status:** Patients must have an Eastern Cooperative Oncology Group performance status of 2 or lower.  **Life Expectancy:** Patients must have a life expectancy of greater than 24 weeks. | Previous splenectomy, spleen irradiation within 3 months before treatment.  Use of investigational agents or hematopoietic growth factors within 28 days before randomization.  Unresolved non-hematological toxic effects, grade 2 or higher peripheral neuropathy, or uncontrolled intercurrent illness reducing study compliance. |
| PERSIST-1 | **Age:** Patients must be aged 18 years or older.  **Diagnosis:** Confirmed diagnosis of primary myelofibrosis, post-essential thrombocythemia myelofibrosis, or post-polycythemia vera myelofibrosis, locally confirmed via bone marrow biopsy.  **Disease Risk:** Patients must have intermediate-risk or high-risk disease according to the Dynamic International Prognostic Scoring System (DIPSS).  **Splenomegaly:** Patients must have a palpable spleen at least 5 cm below the left costal margin.  **Symptoms/Performance Status:** Patients must either have symptoms as per the Myeloproliferative Neoplasm Symptom Assessment Form Total Symptom Score (MPN-SAF TSS) or have an Eastern Cooperative Oncology Group performance status of 0 to 3.  Peripheral Blast Count: Peripheral blast count must be lower than 10%.  **Hematologic and Organ Function:** Patients must have adequate hematologic parameters and organ function.  **Life Expectancy:** Patients must have a life expectancy of 6 months or more.  **Prior Medical History:** Patients must not have undergone prior splenectomy, allogeneic stem cell transplantation, or treatment with JAK2 inhibitors. | Exclusion of patients with specific medical conditions, including inflammatory or chronic functional bowel disorders, uncontrolled cardiovascular  disease, ongoing cardiac dysrhythmias of grade 3 or higher, prolonged corrected QT interval, HIV, active hepatitis A, B, or C, and pregnancy. |
| PERSIST-2 | **Diagnosis:** Adult patients with primary or secondary myelofibrosis.  **Risk Score:** Intermediate-1, intermediate-2, or high-risk disease by the Dynamic International Prognostic Scoring System (DIPSS).  **Platelet Count:** Platelet count less than or equal to 100 × 10^9 /L.  **Splenomegaly:** Palpable splenomegaly 5 cm or larger below the left costal margin.  **Symptom Score:** Total symptom score (TSS) greater than or equal to 13 on the Myeloproliferative Neoplasm Symptom Assessment Form Total Symptom Score (MPN-SAF TSS 2.0).  **Performance Status:** Eastern Cooperative Oncology Group performance status 0 to 3.  **Peripheral Blood Blast Count:** Peripheral blood blast count less than 10%.  **Absolute Neutrophil Count:** Absolute neutrophil count greater than 0.5 × 10^9 /L.  **Organ Function:** Adequate liver and renal function.  **Life Expectancy:** Life expectancy 6 months or longer.  **Prior Treatment:** Prior treatment with 1 or 2 other JAK inhibitors was allowed. | Bleeding: Active bleeding requiring hospitalization during screening. Cardiac Abnormalities: Significant cardiac abnormalities including recent history of myocardial infarction, severe/unstable angina, symptomatic congestive heart failure, ongoing grade ≥3 dysrhythmias, prolonged QTc. Other Exclusion Criteria: Additional details on exclusion criteria are available in the electronic methods provided in the supplementary material. |
| JAKARTA | **Age:** Patients were at least 18 years old.  **Diagnosis:** Patients with a diagnosis of primary myelofibrosis (MF), post-polycythemia vera (PV) MF, or post-essential thrombocythemia (ET) MF.  **Disease Risk:** High-risk or intermediate-2 risk disease according to 2008 World Health Organization and modified International Working Group for Myelofibrosis Research and Treatment criteria.  **Life Expectancy:** At least 6 months.  **Performance Status:** Eastern Cooperative Oncology Group performance status of 0 to 2.  **Splenomegaly:** Palpable splenomegaly (≥5 cm below the left costal margin).  **Platelet Count:** Platelet count at least 50 × 10^3 /μL. |  |
| SAR302503 | **Age:** Patients must be at least 18 years of age.  **Diagnosis:** Patients diagnosed with primary myelofibrosis (PMF), post-polycythemia vera myelofibrosis (post-PV MF), or post-essential thrombocythemia myelofibrosis (post-ET MF) according to the 2008 WHO criteria.  **Disease Risk:** Patients with intermediate-risk level 2 or high-risk myelofibrosis according to the International Working Group-Myeloproliferative Neoplasms Research and Treatment criteria.  **Performance Status:** Eastern Collaborative Oncology Group performance status ≤2. Splenomegaly: Palpable splenomegaly ≥5 cm below the costal margin. Platelet Counts: Platelet counts ≥50 × 10^9 /L.  **JAK2 Mutational Status:** Patients were enrolled regardless of JAK2 mutational status. | Splenectomy: Patients who have undergone splenectomy.  Previous Treatment: Patients who have received previous treatment with a JAK2 inhibitor or any chemotherapy at any time before study entry.  Immunomodulatory Therapy: Patients who have received immunomodulatory therapy or immunosuppressive therapy within 14 days before treatment. |
| PAC203 | **Age:** Adult patients.  **Diagnosis:** Patients with primary or secondary myelofibrosis.  **Disease Risk:** Patients with intermediate-1, intermediate-2, or high-risk disease according to the Dynamic International Prognostic Scoring System.  **Intolerance to Ruxolitinib:** Patients intolerant of ruxolitinib treatment, defined as treatment for 28 days or more, with complications such as a requirement for red blood cell transfusion, grade ≥3 anemia, thrombocytopenia, hematoma, and/or hemorrhage while being treated with a dosage of <20 mg twice per day, or resistant to ruxolitinib (defined as treatment for 3 or more months with <10% spleen volume reduction or <30% decrease in spleen length or regrowth to these parameters).  **Splenomegaly:** Splenomegaly ≥5 cm below the left costal margin.  **Symptom Score:** Total symptom score (TSS) ≥10 on the Myeloproliferative Neoplasm Symptom Assessment Form Total Symptom Score (MPN-SAF TSS 2.0, 7-component version), or single symptom score ≥5 or 2 scores ≥3, including symptoms of left upper quadrant pain, bone pain, itching, or night sweats.  Performance Status: Eastern Cooperative Oncology Group performance status of 0 to 2.  **Peripheral Blast Count:** Peripheral blast count <10%. Absolute **Neutrophil Count:** Absolute neutrophil count >0.5 × 10^9 /L.  **Organ Function:** Adequate liver, renal, and coagulation parameters, with a left ventricular ejection fraction ≥45%.  **Life Expectancy:** Life expectancy ≥6 months. | Grade ≥2 bleeding events within the previous 3 months.  Treatment with anticoagulation or antiplatelet agents (except for aspirin at dosages ≤100 mg per day) within the past 14 days.  New York Heart Association Class ≥2 heart failure.  Grade ≥2 cardiac conditions within the previous 6 months.  QTc prolongation >450 msec during screening.  Treatment with agents that prolong the QT interval within the past 14 days. |

1. **Exposed JAKi subgroup analysis**

According to whether the inclusion criteria expose JAKi, we conducted sub-group analyses. Among the studies with inclusion criteria that previously exposed JAKi are SIMPLIFY-2, PERSIST-2, PAC203, including six treatment modalities: BAT, MMB200Qd, PACPAC400mg Qd, PAC200mg Bid, PAC100mg Bid, and PAC100mg Qd. The results of the sub-group analyses are as follows:


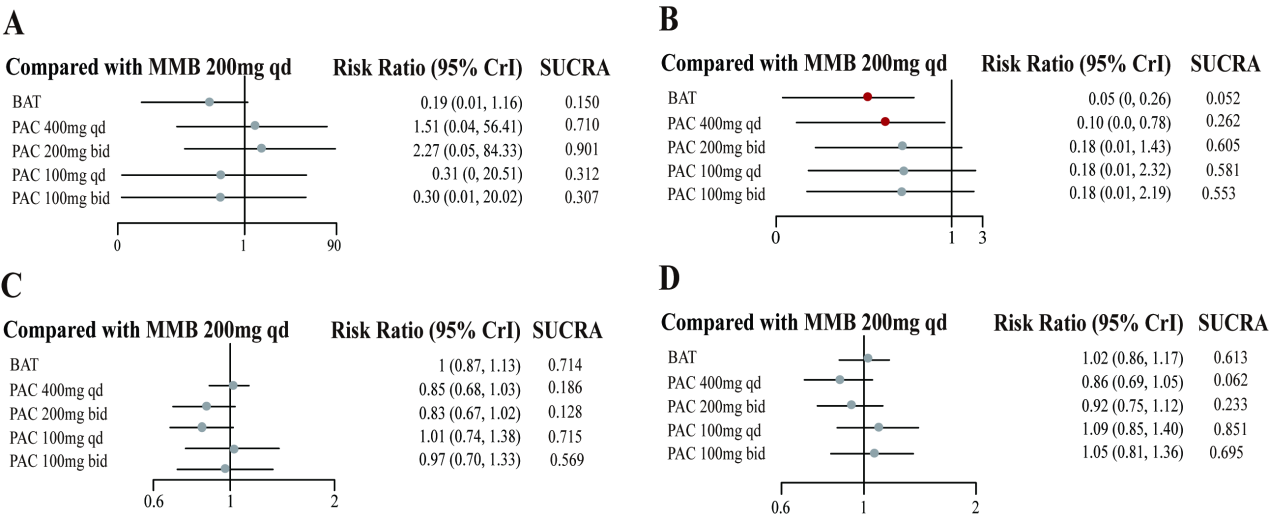


Supplementary Figure. Forest plot of exposed JAKi analysis: Estimates of risk in the intention-to-treat population for (A) SVR, in the per protocol population for (B) TSSR, in the intention-to-treat population for (C) grade 3/4 anemia events, and for (D) grade 3/4 thrombocytopenia events.

Among the studies with inclusion criteria that previously did not expose JAKi are COMFORT-I, SIMPLIFY-1, JAKARTA, SAR302503, PERSIST-1, COMFORT-II, including six treatment modalities: RUX, PLB, MMB200Qd, FED300Qd, FED400Qd, and FED500Qd. The results of the sub-group analyses are as follows:


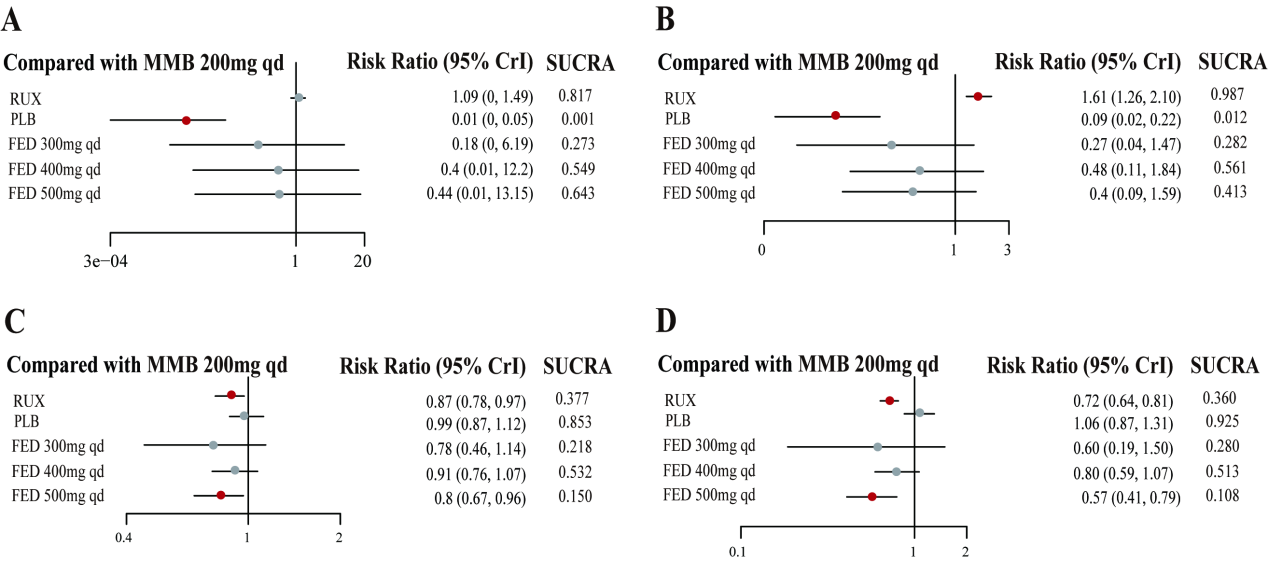


Supplementary Figure. Forest plot of unexposed JAKi analysis: Estimates of risk in the intention-to-treat population for (A) SVR, in the per protocol population for (B) TSSR, in the intention-to-treat population for (C) grade 3/4 anemia events, and for (D) grade 3/4 thrombocytopenia events.

According to the results, among patients previously exposed to JAKi, none of the JAKi therapies showed efficacy and safety superior to MMB. Meanwhile, among patients not previously exposed to JAKi, RUX demonstrated superior efficacy compared to MMB, but in terms of hematologic safety, MMB outperformed RUX. Additionally, other JAKi therapies were inferior to MMB in terms of efficacy. These findings are consistent with the conclusions of this study.

1. **Total symptom score (TSS) Subgroup Analysis**

The RCTs employing the MF-TSS assessment tool include COMFORT-I and JAKARTA, involving four therapeutic modalities: ruxolitinib (RUX), placebo (PLB), and fedratinib at doses of 400 mg once daily (qd) and 500 mg qd. RCTs utilizing the MPN-TSS assessment tool encompass SIMPLIFY-1, SIMPLIFY-2, and PERSIST-1, involving RUX, BAT, momelotinib at 200 mg qd, and pacritinib at 400 mg qd as the therapeutic strategies. Subgroup analysis based on different assessment tools revealed that the symptom relief efficacy of fedratinib 400 mg qd was comparable to RUX, followed by momelotinib. Conversely, PAC, BAT, and PLB were significantly inferior to RUX. These outcomes are in general agreement with the conclusions.


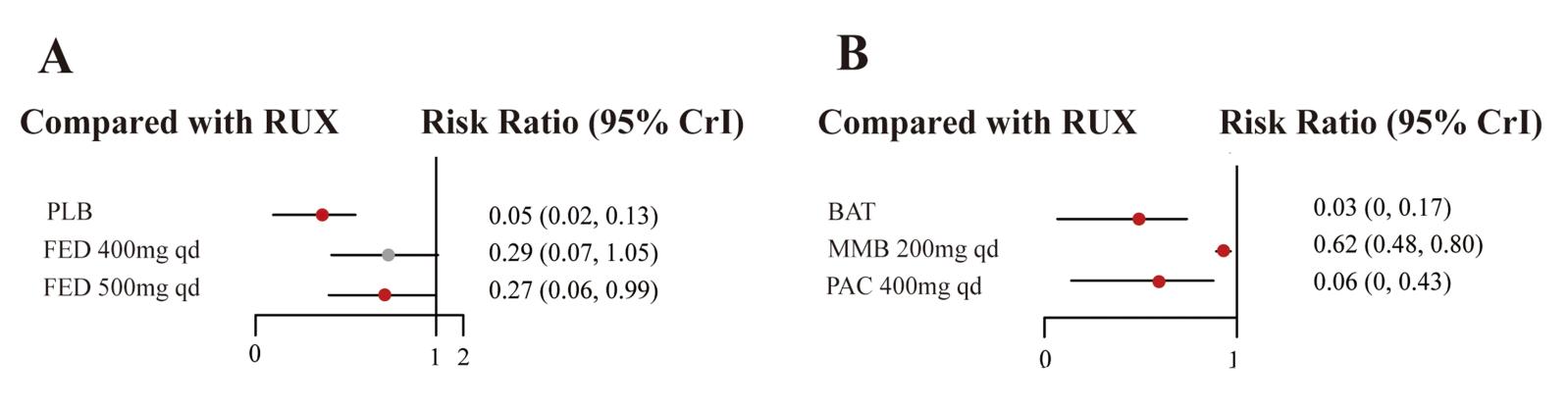


**6. Heterogeneity analysis**

**6.1 SVR**


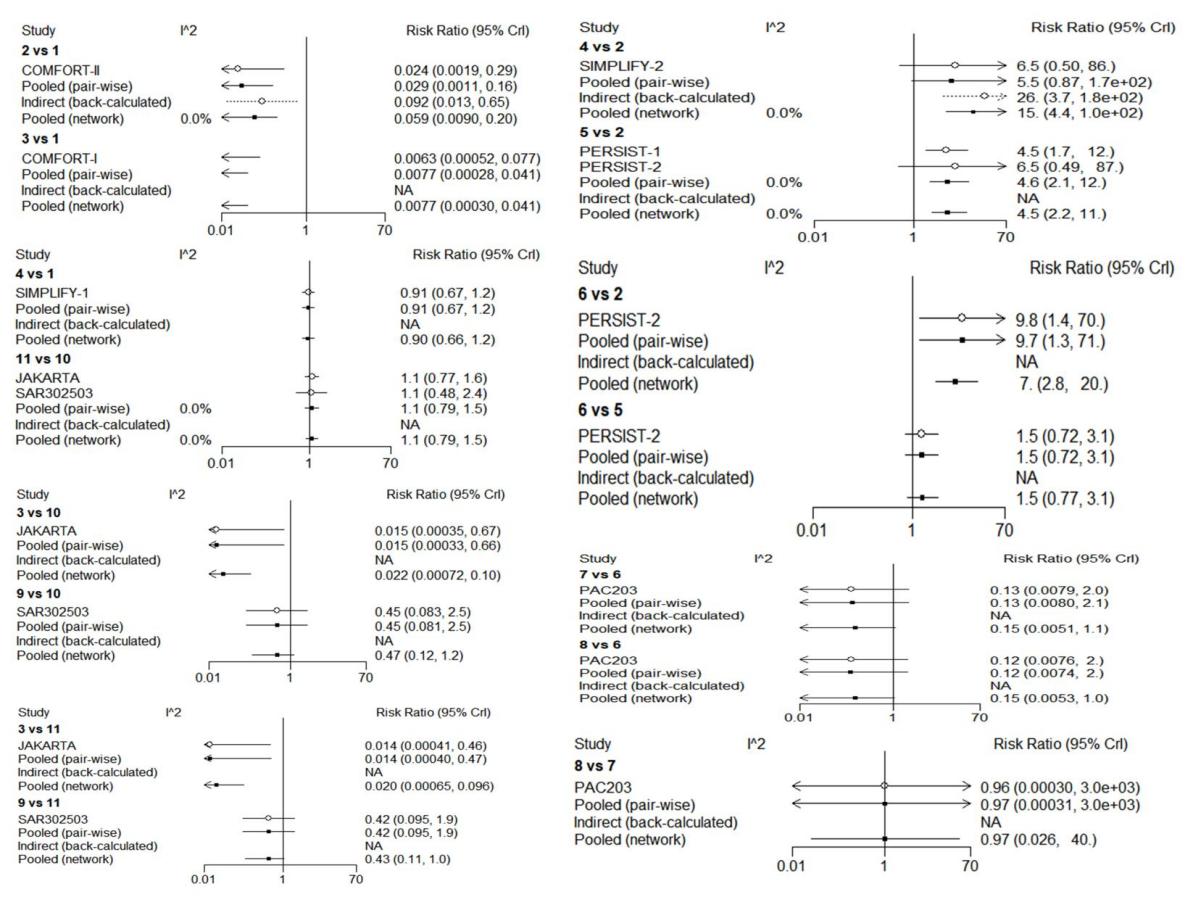


**6.2 TSSR**


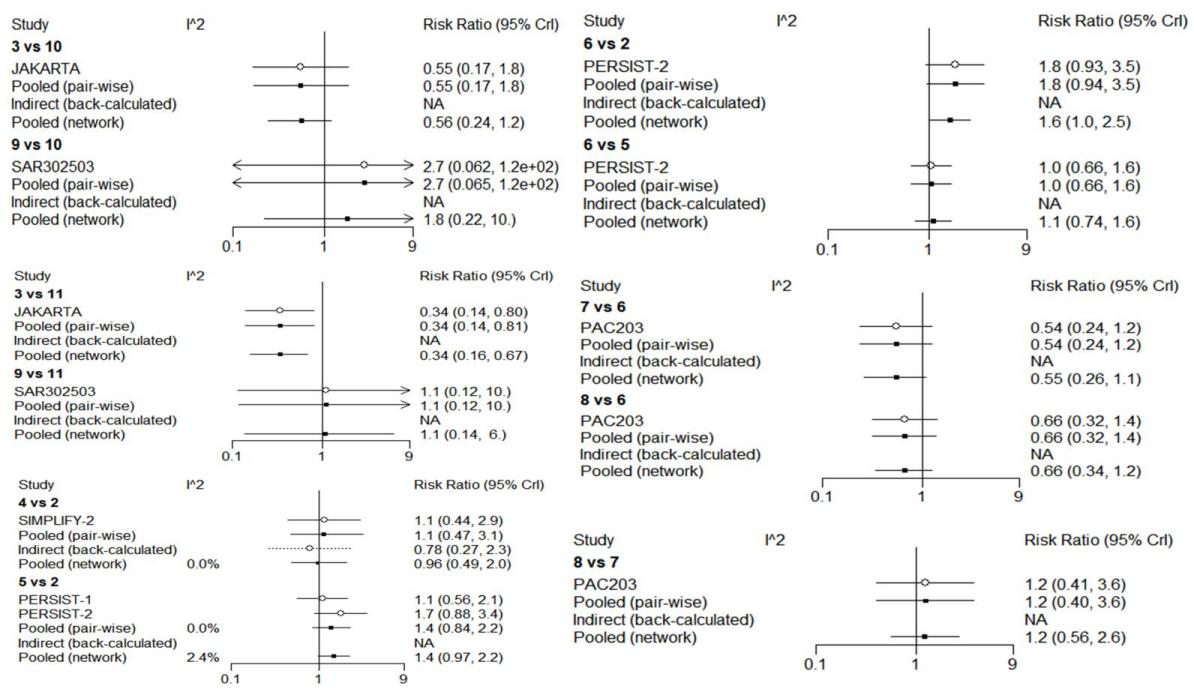


**6.3 Anemia (grade 3or4)**


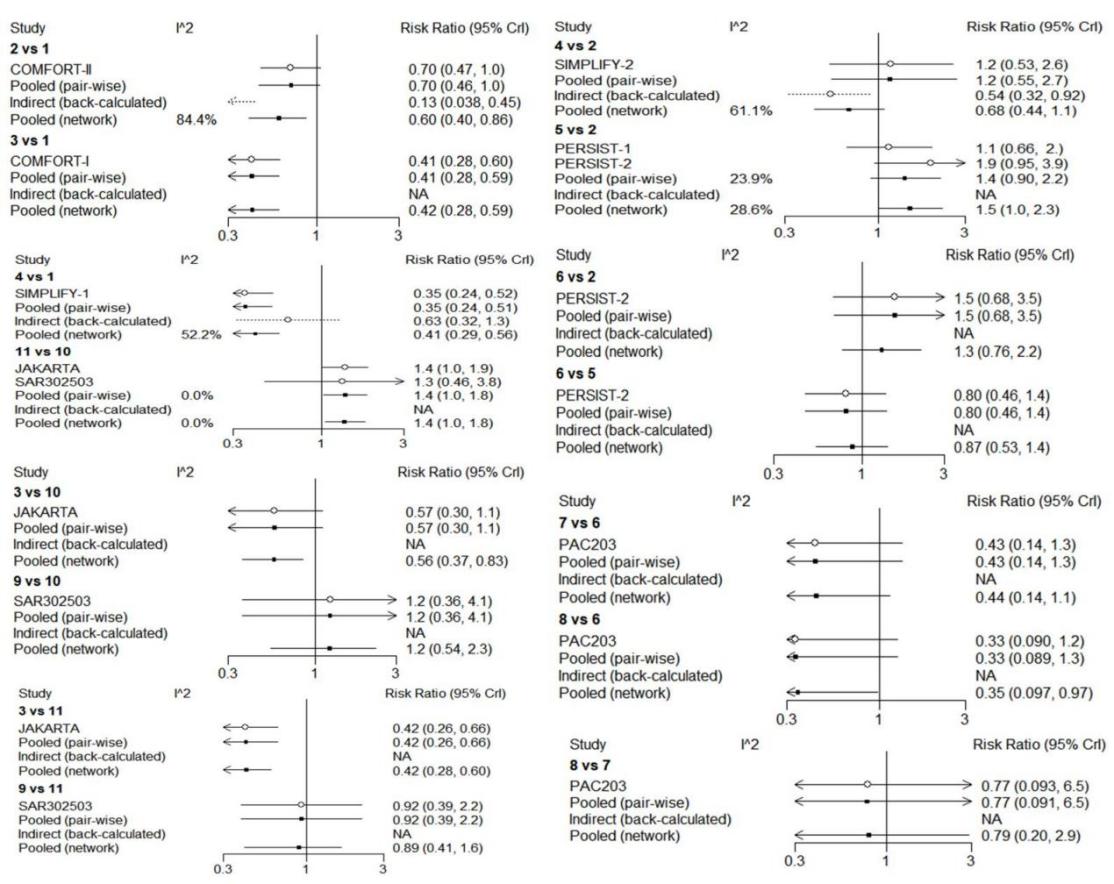


**6.4 Thrombocytopenia (grade 3or4)**

**
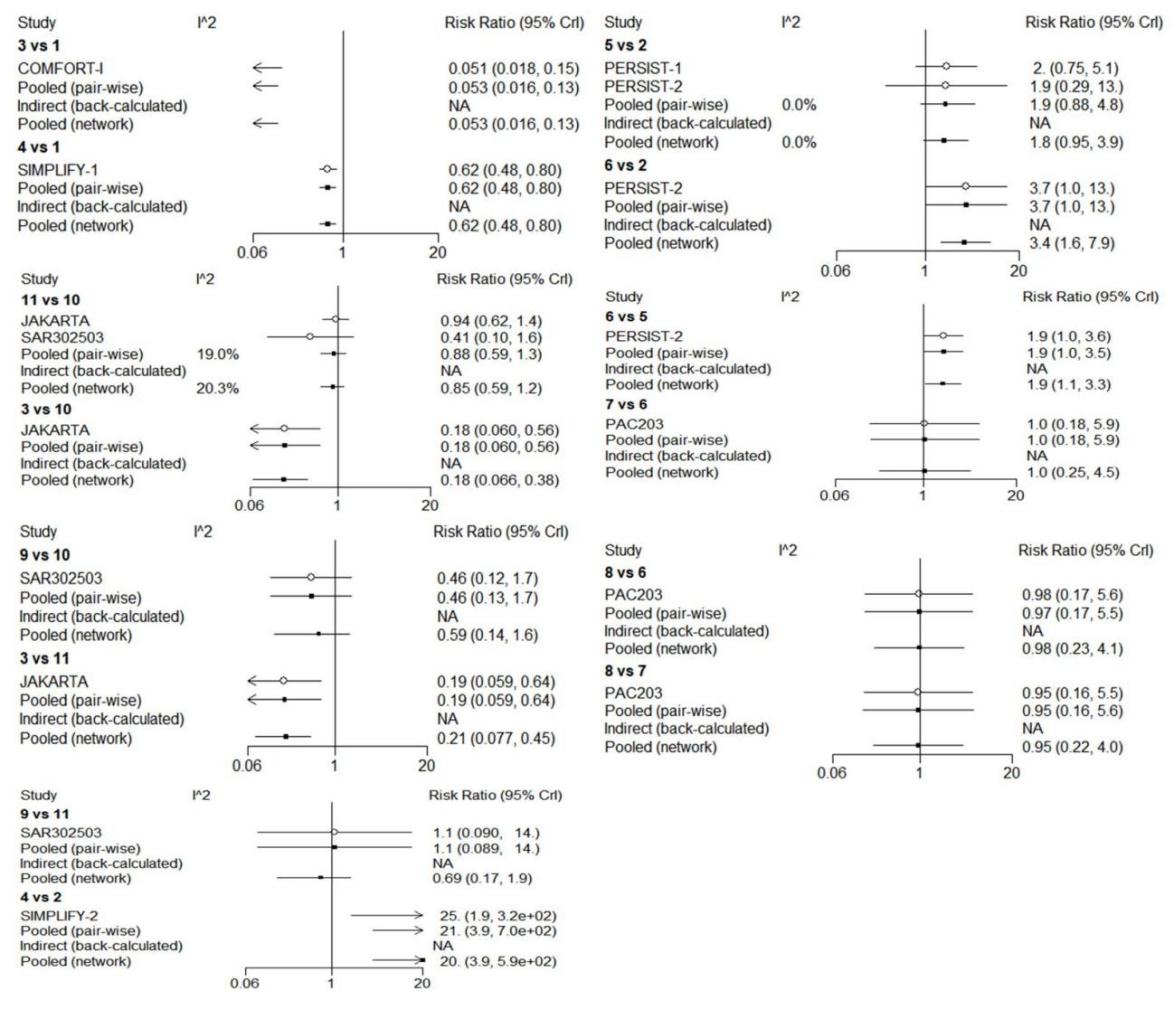
**

**7.Consistency analysis**

**7.1 SVR**

**
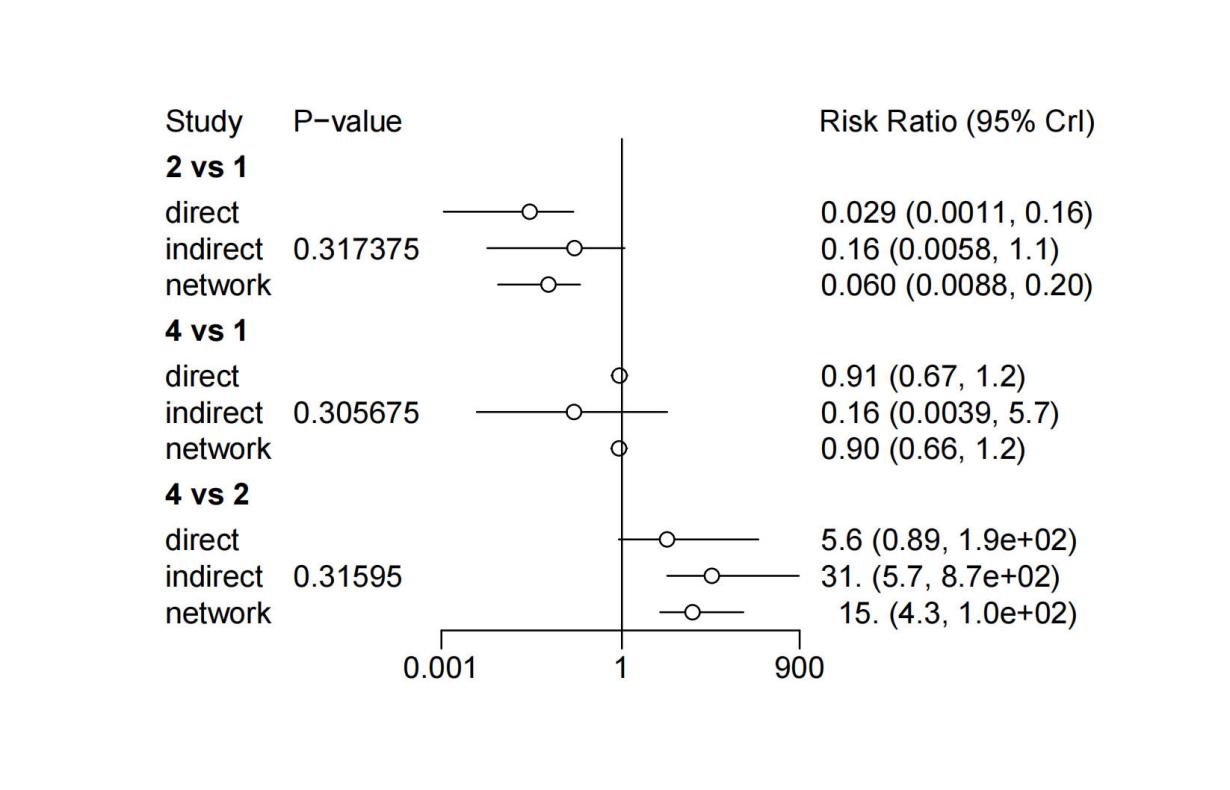
**

**7.2 Anemia (grade 3or4)**

**
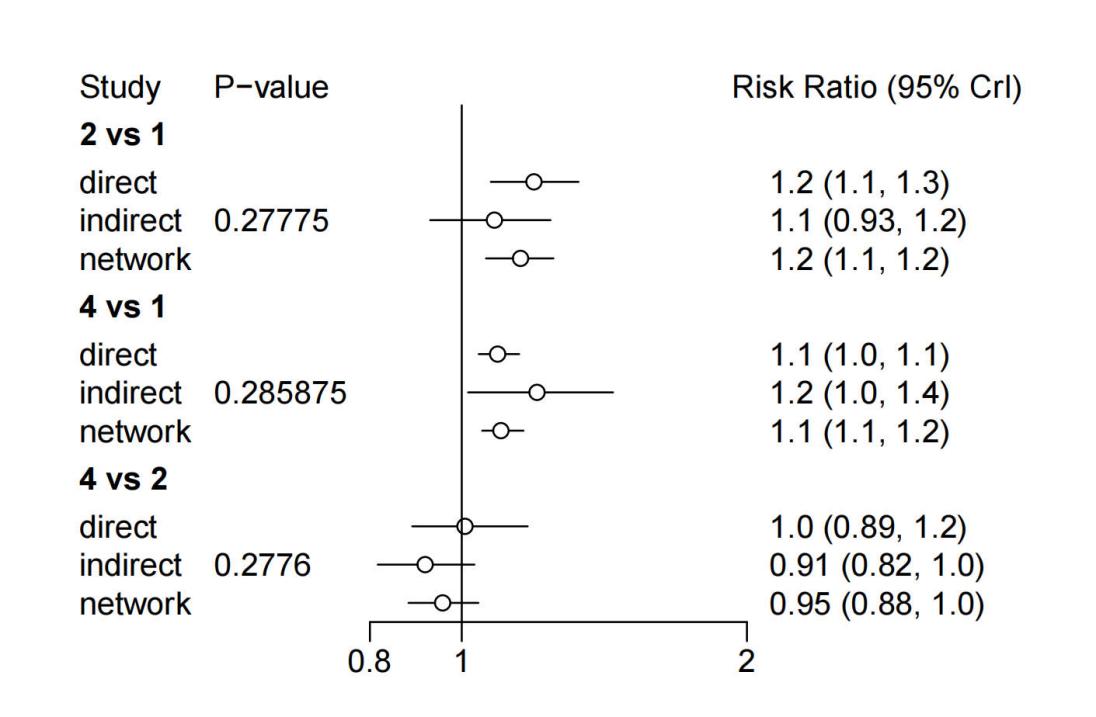
**

**7.3 Thrombocytopenia (grade 3or4)**

**
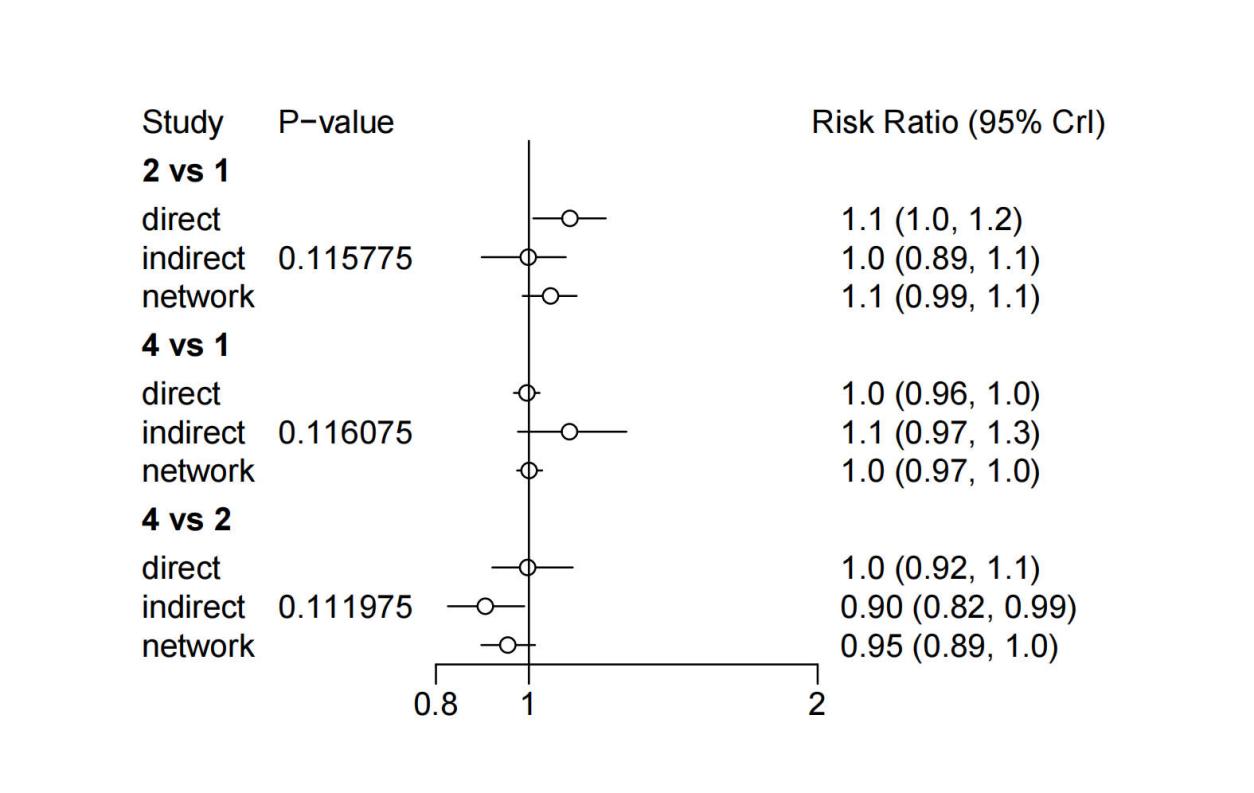
**

**8.CINeMA confidence rating result**

**8.1 CINeMA confidence rating result of SVR**

| **Comparison** | **Within-study bias** | **Reporting bias** | **Indirectness** | **Imprecision** | **Heterogeneity** | **Incoherence** | **Confidence rating** | **Reason(s) for downgrading** |
| --- | --- | --- | --- | --- | --- | --- | --- | --- |
| BAT:MMB200Qd | No concerns | Low risk | No concerns | No concerns | Some concerns | No concerns | High | "Heterogeneity" |
| BAT:PAC200Bid | No concerns | Low risk | No concerns | No concerns | Some concerns | No concerns | High | "Heterogeneity" |
| BAT:PAC400Qd | No concerns | Low risk | No concerns | No concerns | Some concerns | No concerns | High | "Heterogeneity" |
| BAT:RUX | No concerns | Low risk | No concerns | No concerns | Some concerns | No concerns | High | "Heterogeneity" |
| FED300Qd:FED400Qd | No concerns | Low risk | No concerns | No concerns | Some concerns | No concerns | High | "Heterogeneity" |
| FED300Qd:FED500Qd | No concerns | Low risk | No concerns | No concerns | Some concerns | No concerns | High | "Heterogeneity" |
| FED400Qd:FED500Qd | No concerns | Low risk | No concerns | No concerns | No concerns | No concerns | High |  |
| FED400Qd:PLB | No concerns | Low risk | No concerns | No concerns | Some concerns | No concerns | High | "Heterogeneity" |
| FED500Qd:PLB | No concerns | Low risk | No concerns | No concerns | Some concerns | No concerns | High | "Heterogeneity" |
| MMB200Qd:RUX | No concerns | Low risk | No concerns | No concerns | No concerns | No concerns | High |  |
| PAC100Bid:PAC100Qd | No concerns | Low risk | No concerns | Major concerns | No concerns | No concerns | Moderate | "Imprecision" |
| PAC100Bid:PAC200Bid | No concerns | Low risk | No concerns | Some concerns | Some concerns | No concerns | High | "Imprecision",  "Heterogeneity" |
| PAC100Qd:PAC200Bid | No concerns | Low risk | No concerns | Some concerns | Some concerns | No concerns | High | "Imprecision",  "Heterogeneity" |
| PAC200Bid:PAC400Qd | No concerns | Low risk | No concerns | No concerns | No concerns | No concerns | High |  |
| PLB:RUX | No concerns | Low risk | No concerns | No concerns | No concerns | No concerns | High |  |
| BAT:FED300Qd | No concerns | Low risk | No concerns | Major concerns | No concerns | Some concerns | Moderate | "Imprecision",  "Incoherence" |
| BAT:FED400Qd | No concerns | Low risk | No concerns | Some concerns | Some concerns | Some concerns | Moderate | "Imprecision",  "Heterogeneity",  "Incoherence" |
| BAT:FED500Qd | No concerns | Low risk | No concerns | Some concerns | Some concerns | Some concerns | Moderate | "Imprecision",  "Heterogeneity",  "Incoherence" |
| BAT:PAC100Bid | No concerns | Low risk | No concerns | Some concerns | Some concerns | Some concerns | Moderate | "Imprecision",  "Heterogeneity",  "Incoherence" |
| BAT:PAC100Qd | No concerns | Low risk | No concerns | Some concerns | Some concerns | Some concerns | Moderate | "Imprecision",  "Heterogeneity",  "Incoherence" |
| BAT:PLB | No concerns | Low risk | No concerns | Some concerns | Some concerns | Some concerns | Moderate | "Imprecision",  "Heterogeneity",  "Incoherence" |
| FED300Qd:MMB200Qd | No concerns | Low risk | No concerns | Some concerns | Some concerns | Some concerns | Moderate | "Imprecision",  "Heterogeneity",  "Incoherence" |
| FED300Qd:PAC100Bid | No concerns | Low risk | No concerns | Major concerns | No concerns | Some concerns | Moderate | "Imprecision",  "Incoherence" |
| FED300Qd:PAC100Qd | No concerns | Low risk | No concerns | Major concerns | No concerns | Some concerns | Moderate | "Imprecision",  "Incoherence" |
| FED300Qd:PAC200Bid | No concerns | Low risk | No concerns | Major concerns | No concerns | Some concerns | Moderate | "Imprecision",  "Incoherence" |
| FED300Qd:PAC400Qd | No concerns | Low risk | No concerns | Major concerns | No concerns | Some concerns | Moderate | "Imprecision",  "Incoherence" |
| FED300Qd:PLB | No concerns | Low risk | No concerns | No concerns | Some concerns | Some concerns | High | "Heterogeneity",  "Incoherence" |
| FED300Qd:RUX | No concerns | Low risk | No concerns | Some concerns | Some concerns | Some concerns | Moderate | "Imprecision",  "Heterogeneity",  "Incoherence" |
| FED400Qd:MMB200Qd | No concerns | Low risk | No concerns | Some concerns | Some concerns | Some concerns | Moderate | "Imprecision",  "Heterogeneity",  "Incoherence" |
| FED400Qd:PAC100Bid | No concerns | Low risk | No concerns | Major concerns | No concerns | Some concerns | Moderate | "Imprecision",  "Incoherence" |
| FED400Qd:PAC100Qd | No concerns | Low risk | No concerns | Major concerns | No concerns | Some concerns | Moderate | "Imprecision",  "Incoherence" |
| FED400Qd:PAC200Bid | No concerns | Low risk | No concerns | Major concerns | No concerns | Some concerns | Moderate | "Imprecision",  "Incoherence" |
| FED400Qd:PAC400Qd | No concerns | Low risk | No concerns | Major concerns | No concerns | Some concerns | Moderate | "Imprecision",  "Incoherence" |
| FED400Qd:RUX | No concerns | Low risk | No concerns | Some concerns | Some concerns | Some concerns | Moderate | "Imprecision",  "Heterogeneity",  "Incoherence" |
| FED500Qd:MMB200Qd | No concerns | Low risk | No concerns | Some concerns | Some concerns | Some concerns | Moderate | "Imprecision",  "Heterogeneity",  "Incoherence" |
| FED500Qd:PAC100Bid | No concerns | Low risk | No concerns | Major concerns | No concerns | Some concerns | Moderate | "Imprecision",  "Incoherence" |
| FED500Qd:PAC100Qd | No concerns | Low risk | No concerns | Major concerns | No concerns | Some concerns | Moderate | "Imprecision",  "Incoherence" |
| FED500Qd:PAC200Bid | No concerns | Low risk | No concerns | Major concerns | No concerns | Some concerns | Moderate | "Imprecision",  "Incoherence" |
| FED500Qd:PAC400Qd | No concerns | Low risk | No concerns | Major concerns | No concerns | Some concerns | Moderate | "Imprecision",  "Incoherence" |
| FED500Qd:RUX | No concerns | Low risk | No concerns | Some concerns | Some concerns | Some concerns | Moderate | "Imprecision",  "Heterogeneity",  "Incoherence" |
| MMB200Qd:PAC100Bid | No concerns | Low risk | No concerns | Some concerns | Some concerns | Some concerns | Moderate | "Imprecision",  "Heterogeneity",  "Incoherence" |
| MMB200Qd:PAC100Qd | No concerns | Low risk | No concerns | Some concerns | Some concerns | Some concerns | Moderate | "Imprecision",  "Heterogeneity",  "Incoherence" |
| MMB200Qd:PAC200Bid | No concerns | Low risk | No concerns | No concerns | Major concerns | Some concerns | Moderate | "Heterogeneity",  "Incoherence" |
| MMB200Qd:PAC400Qd | No concerns | Low risk | No concerns | Some concerns | Some concerns | Some concerns | Moderate | "Imprecision",  "Heterogeneity",  "Incoherence" |
| MMB200Qd:PLB | No concerns | Low risk | No concerns | No concerns | No concerns | Some concerns | High | "Incoherence" |
| PAC100Bid:PAC400Qd | No concerns | Low risk | No concerns | Some concerns | Some concerns | Some concerns | Moderate | "Imprecision",  "Heterogeneity",  "Incoherence" |
| PAC100Bid:PLB | No concerns | Low risk | No concerns | Some concerns | Some concerns | Some concerns | Moderate | "Imprecision",  "Heterogeneity",  "Incoherence" |
| PAC100Bid:RUX | No concerns | Low risk | No concerns | Some concerns | Some concerns | Some concerns | Moderate | "Imprecision",  "Heterogeneity",  "Incoherence" |
| PAC100Qd:PAC400Qd | No concerns | Low risk | No concerns | Some concerns | Some concerns | Some concerns | Moderate | "Imprecision",  "Heterogeneity",  "Incoherence" |
| PAC100Qd:PLB | No concerns | Low risk | No concerns | Some concerns | Some concerns | Some concerns | Moderate | "Imprecision",  "Heterogeneity",  "Incoherence" |
| PAC100Qd:RUX | No concerns | Low risk | No concerns | Some concerns | Some concerns | Some concerns | Moderate | "Imprecision",  "Heterogeneity",  "Incoherence" |
| PAC200Bid:PLB | No concerns | Low risk | No concerns | No concerns | Some concerns | Some concerns | High | "Heterogeneity",  "Incoherence" |
| PAC200Bid:RUX | No concerns | Low risk | No concerns | No concerns | Major concerns | Some concerns | Moderate | "Heterogeneity",  "Incoherence" |
| PAC400Qd:PLB | No concerns | Low risk | No concerns | No concerns | Some concerns | Some concerns | High | "Heterogeneity",  "Incoherence" |
| PAC400Qd:RUX | No concerns | Low risk | No concerns | Some concerns | Some concerns | Some concerns | Moderate | "Imprecision",  "Heterogeneity",  "Incoherence" |

**8.2 CINeMA confidence rating result of TSSR**

| **Comparison** | **Within-study bias** | **Reporting bias** | **Indirectness** | **Imprecision** | **Heterogeneity** | **Incoherence** | **Confidence rating** | **Reason(s) for downgrading** |
| --- | --- | --- | --- | --- | --- | --- | --- | --- |
| BAT:MMB200Qd | No concerns | Low risk | No concerns | No concerns | Some concerns | No concerns | High | "Heterogeneity" |
| BAT:PAC200Bid | No concerns | Low risk | No concerns | No concerns | Some concerns | No concerns | High | "Heterogeneity" |
| BAT:PAC400Qd | No concerns | Low risk | No concerns | No concerns | Some concerns | No concerns | High | "Heterogeneity" |
| FED300Qd:FED400Qd | No concerns | Low risk | No concerns | No concerns | Some concerns | No concerns | High | "Heterogeneity" |
| FED300Qd:FED500Qd | No concerns | Low risk | No concerns | No concerns | Some concerns | No concerns | High | "Heterogeneity" |
| FED400Qd:FED500Qd | No concerns | Low risk | No concerns | No concerns | Some concerns | No concerns | High | "Heterogeneity" |
| FED400Qd:PLB | No concerns | Low risk | No concerns | No concerns | No concerns | No concerns | High |  |
| FED500Qd:PLB | No concerns | Low risk | No concerns | No concerns | Some concerns | No concerns | High | "Heterogeneity" |
| MMB200Qd:RUX | No concerns | Low risk | No concerns | No concerns | Some concerns | No concerns | High | "Heterogeneity" |
| PAC100Bid:PAC100Qd | No concerns | Low risk | No concerns | No concerns | No concerns | No concerns | High |  |
| PAC100Bid:PAC200Bid | No concerns | Low risk | No concerns | Major concerns | No concerns | No concerns | Moderate | "Imprecision" |
| PAC100Qd:PAC200Bid | No concerns | Low risk | No concerns | Some concerns | Some concerns | No concerns | High | "Imprecision",  "Heterogeneity" |
| PAC200Bid:PAC400Qd | No concerns | Low risk | No concerns | Some concerns | Some concerns | No concerns | High | "Imprecision",  "Heterogeneity" |
| PLB:RUX | No concerns | Low risk | No concerns | No concerns | No concerns | No concerns | High |  |
| BAT:FED300Qd | No concerns | Low risk | No concerns | No concerns | No concerns | No concerns | High |  |
| BAT:FED400Qd | No concerns | Low risk | No concerns | Major concerns | No concerns | Some concerns | Moderate | "Imprecision",  "Incoherence" |
| BAT:FED500Qd | No concerns | Low risk | No concerns | Some concerns | Some concerns | Some concerns | Moderate | "Imprecision",  "Heterogeneity",  "Incoherence" |
| BAT:PAC100Bid | No concerns | Low risk | No concerns | Some concerns | Some concerns | Some concerns | Moderate | "Imprecision",  "Heterogeneity",  "Incoherence" |
| BAT:PAC100Qd | No concerns | Low risk | No concerns | Some concerns | Some concerns | Some concerns | Moderate | "Imprecision",  "Heterogeneity",  "Incoherence" |
| BAT:PLB | No concerns | Low risk | No concerns | Some concerns | Some concerns | Some concerns | Moderate | "Imprecision",  "Heterogeneity",  "Incoherence" |
| BAT:RUX | No concerns | Low risk | No concerns | Some concerns | Some concerns | Some concerns | Moderate | "Imprecision",  "Heterogeneity",  "Incoherence" |
| FED300Qd:MMB200Qd | No concerns | Low risk | No concerns | Some concerns | Some concerns | Some concerns | Moderate | "Imprecision",  "Heterogeneity",  "Incoherence" |
| FED300Qd:PAC100Bid | No concerns | Low risk | No concerns | Major concerns | No concerns | Some concerns | Moderate | "Imprecision",  "Incoherence" |
| FED300Qd:PAC100Qd | No concerns | Low risk | No concerns | Major concerns | No concerns | Some concerns | Moderate | "Imprecision",  "Incoherence" |
| FED300Qd:PAC200Bid | No concerns | Low risk | No concerns | Major concerns | No concerns | Some concerns | Moderate | "Imprecision",  "Incoherence" |
| FED300Qd:PAC400Qd | No concerns | Low risk | No concerns | Major concerns | No concerns | Some concerns | Moderate | "Imprecision",  "Incoherence" |
| FED300Qd:PLB | No concerns | Low risk | No concerns | No concerns | Some concerns | Some concerns | High | "Heterogeneity",  "Incoherence" |
| FED300Qd:RUX | No concerns | Low risk | No concerns | Some concerns | Some concerns | Some concerns | Moderate | "Imprecision",  "Heterogeneity",  "Incoherence" |
| FED400Qd:MMB200Qd | No concerns | Low risk | No concerns | Some concerns | Some concerns | Some concerns | Moderate | "Imprecision",  "Heterogeneity",  "Incoherence" |
| FED400Qd:PAC100Bid | No concerns | Low risk | No concerns | Major concerns | No concerns | Some concerns | Moderate | "Imprecision",  "Incoherence" |
| FED400Qd:PAC100Qd | No concerns | Low risk | No concerns | Major concerns | No concerns | Some concerns | Moderate | "Imprecision",  "Incoherence" |
| FED400Qd:PAC200Bid | No concerns | Low risk | No concerns | Major concerns | No concerns | Some concerns | Moderate | "Imprecision",  "Incoherence" |
| FED400Qd:PAC400Qd | No concerns | Low risk | No concerns | Major concerns | No concerns | Some concerns | Moderate | "Imprecision",  "Incoherence" |
| FED400Qd:RUX | No concerns | Low risk | No concerns | Some concerns | Some concerns | Some concerns | Moderate | "Imprecision",  "Heterogeneity",  "Incoherence" |
| FED500Qd:MMB200Qd | No concerns | Low risk | No concerns | Some concerns | Some concerns | Some concerns | Moderate | "Imprecision",  "Heterogeneity",  "Incoherence" |
| FED500Qd:PAC100Bid | No concerns | Low risk | No concerns | Major concerns | No concerns | Some concerns | Moderate | "Imprecision",  "Incoherence" |
| FED500Qd:PAC100Qd | No concerns | Low risk | No concerns | Major concerns | No concerns | Some concerns | Moderate | "Imprecision",  "Incoherence" |
| FED500Qd:PAC200Bid | No concerns | Low risk | No concerns | Major concerns | No concerns | Some concerns | Moderate | "Imprecision",  "Incoherence" |
| FED500Qd:PAC400Qd | No concerns | Low risk | No concerns | Major concerns | No concerns | Some concerns | Moderate | "Imprecision",  "Incoherence" |
| FED500Qd:RUX | No concerns | Low risk | No concerns | Some concerns | Some concerns | Some concerns | Moderate | "Imprecision",  "Heterogeneity",  "Incoherence" |
| MMB200Qd:PAC100Bid | No concerns | Low risk | No concerns | Some concerns | Some concerns | Some concerns | Moderate | "Imprecision",  "Heterogeneity",  "Incoherence" |
| MMB200Qd:PAC100Qd | No concerns | Low risk | No concerns | Some concerns | Some concerns | Some concerns | Moderate | "Imprecision",  "Heterogeneity",  "Incoherence" |
| MMB200Qd:PAC200Bid | No concerns | Low risk | No concerns | No concerns | Major concerns | Some concerns | Moderate | "Heterogeneity",  "Incoherence" |
| MMB200Qd:PAC400Qd | No concerns | Low risk | No concerns | Some concerns | Some concerns | Some concerns | Moderate | "Imprecision",  "Heterogeneity",  "Incoherence" |
| MMB200Qd:PLB | No concerns | Low risk | No concerns | No concerns | No concerns | Some concerns | High | "Incoherence" |
| PAC100Bid:PAC400Qd | No concerns | Low risk | No concerns | Some concerns | Some concerns | Some concerns | Moderate | "Imprecision",  "Heterogeneity",  "Incoherence" |
| PAC100Bid:PLB | No concerns | Low risk | No concerns | Some concerns | Some concerns | Some concerns | Moderate | "Imprecision",  "Heterogeneity",  "Incoherence" |
| PAC100Bid:RUX | No concerns | Low risk | No concerns | Some concerns | Some concerns | Some concerns | Moderate | "Imprecision",  "Heterogeneity",  "Incoherence" |
| PAC100Qd:PAC400Qd | No concerns | Low risk | No concerns | Some concerns | Some concerns | Some concerns | Moderate | "Imprecision",  "Heterogeneity",  "Incoherence" |
| PAC100Qd:PLB | No concerns | Low risk | No concerns | Some concerns | Some concerns | Some concerns | Moderate | "Imprecision",  "Heterogeneity",  "Incoherence" |
| PAC100Qd:RUX | No concerns | Low risk | No concerns | Some concerns | Some concerns | Some concerns | Moderate | "Imprecision",  "Heterogeneity",  "Incoherence" |
| PAC200Bid:PLB | No concerns | Low risk | No concerns | No concerns | Some concerns | Some concerns | High | "Heterogeneity",  "Incoherence" |
| PAC200Bid:RUX | No concerns | Low risk | No concerns | No concerns | Major concerns | Some concerns | Moderate | "Heterogeneity",  "Incoherence" |
| PAC400Qd:PLB | No concerns | Low risk | No concerns | No concerns | Some concerns | Some concerns | High | "Heterogeneity",  "Incoherence" |
| PAC400Qd:RUX | No concerns | Low risk | No concerns | Some concerns | Some concerns | Some concerns | Moderate | "Imprecision",  "Heterogeneity",  "Incoherence" |

**8.3 CINeMA confidence rating result of Thrombocytopenia (grade 3or4)**

| **Comparison** | **Within-study bias** | **Reporting bias** | **Indirectness** | **Imprecision** | **Heterogeneity** | **Incoherence** | **Confidence rating** | **Reason(s) for downgrading** |
| --- | --- | --- | --- | --- | --- | --- | --- | --- |
| BAT:MMB200Qd | No concerns | Low risk | No concerns | No concerns | No concerns | No concerns | High |  |
| BAT:PAC200Bid | No concerns | Low risk | No concerns | No concerns | No concerns | No concerns | High |  |
| BAT:PAC400Qd | No concerns | Low risk | No concerns | No concerns | No concerns | No concerns | High |  |
| BAT:RUX | No concerns | Low risk | No concerns | No concerns | No concerns | No concerns | High |  |
| FED300Qd:FED400Qd | No concerns | Low risk | No concerns | No concerns | No concerns | No concerns | High |  |
| FED300Qd:FED500Qd | No concerns | Low risk | No concerns | No concerns | No concerns | No concerns | High |  |
| FED400Qd:FED500Qd | No concerns | Low risk | No concerns | No concerns | No concerns | No concerns | High |  |
| FED400Qd:PLB | No concerns | Low risk | No concerns | No concerns | No concerns | No concerns | High |  |
| FED500Qd:PLB | No concerns | Low risk | No concerns | No concerns | No concerns | No concerns | High |  |
| MMB200Qd:RUX | No concerns | Low risk | No concerns | No concerns | No concerns | No concerns | High |  |
| PAC100Bid:PAC100Qd | No concerns | Low risk | No concerns | No concerns | No concerns | No concerns | High |  |
| PAC100Bid:PAC200Bid | No concerns | Low risk | No concerns | No concerns | No concerns | No concerns | High |  |
| PAC100Qd:PAC200Bid | No concerns | Low risk | No concerns | No concerns | No concerns | No concerns | High |  |
| PAC200Bid:PAC400Qd | No concerns | Low risk | No concerns | No concerns | No concerns | No concerns | High |  |
| PLB:RUX | No concerns | Low risk | No concerns | No concerns | No concerns | No concerns | High |  |
| BAT:FED300Qd | No concerns | Low risk | No concerns | No concerns | No concerns | Some concerns | High |  |
| BAT:FED400Qd | No concerns | Low risk | No concerns | No concerns | No concerns | Some concerns | High |  |
| BAT:FED500Qd | No concerns | Low risk | No concerns | No concerns | No concerns | Some concerns | High |  |
| BAT:PAC100Bid | No concerns | Low risk | No concerns | No concerns | No concerns | Some concerns | High |  |
| BAT:PAC100Qd | No concerns | Low risk | No concerns | No concerns | No concerns | Some concerns | High |  |
| BAT:PLB | No concerns | Low risk | No concerns | No concerns | No concerns | Some concerns | High |  |
| FED300Qd:MMB200Qd | No concerns | Low risk | No concerns | No concerns | No concerns | Some concerns | High |  |
| FED300Qd:PAC100Bid | No concerns | Low risk | No concerns | No concerns | No concerns | Some concerns | High |  |
| FED300Qd:PAC100Qd | No concerns | Low risk | No concerns | No concerns | No concerns | Some concerns | High |  |
| FED300Qd:PAC200Bid | No concerns | Low risk | No concerns | No concerns | No concerns | Some concerns | High |  |
| FED300Qd:PAC400Qd | No concerns | Low risk | No concerns | No concerns | No concerns | Some concerns | High |  |
| FED300Qd:PLB | No concerns | Low risk | No concerns | No concerns | No concerns | Some concerns | High |  |
| FED300Qd:RUX | No concerns | Low risk | No concerns | No concerns | No concerns | Some concerns | High |  |
| FED400Qd:MMB200Qd | No concerns | Low risk | No concerns | No concerns | No concerns | Some concerns | High |  |
| FED400Qd:PAC100Bid | No concerns | Low risk | No concerns | No concerns | No concerns | Some concerns | High |  |
| FED400Qd:PAC100Qd | No concerns | Low risk | No concerns | No concerns | No concerns | Some concerns | High |  |
| FED400Qd:PAC200Bid | No concerns | Low risk | No concerns | No concerns | No concerns | Some concerns | High |  |
| FED400Qd:PAC400Qd | No concerns | Low risk | No concerns | No concerns | No concerns | Some concerns | High |  |
| FED400Qd:RUX | No concerns | Low risk | No concerns | No concerns | No concerns | Some concerns | High |  |
| FED500Qd:MMB200Qd | No concerns | Low risk | No concerns | No concerns | No concerns | Some concerns | High |  |
| FED500Qd:PAC100Bid | No concerns | Low risk | No concerns | No concerns | No concerns | Some concerns | High |  |
| FED500Qd:PAC100Qd | No concerns | Low risk | No concerns | No concerns | No concerns | Some concerns | High |  |
| FED500Qd:PAC200Bid | No concerns | Low risk | No concerns | No concerns | No concerns | Some concerns | High |  |
| FED500Qd:PAC400Qd | No concerns | Low risk | No concerns | No concerns | No concerns | Some concerns | High |  |
| FED500Qd:RUX | No concerns | Low risk | No concerns | No concerns | No concerns | Some concerns | High |  |
| MMB200Qd:PAC100Bid | No concerns | Low risk | No concerns | No concerns | No concerns | Some concerns | High |  |
| MMB200Qd:PAC100Qd | No concerns | Low risk | No concerns | No concerns | No concerns | Some concerns | High |  |
| MMB200Qd:PAC200Bid | No concerns | Low risk | No concerns | No concerns | No concerns | Some concerns | High |  |
| MMB200Qd:PAC400Qd | No concerns | Low risk | No concerns | No concerns | No concerns | Some concerns | High |  |
| MMB200Qd:PLB | No concerns | Low risk | No concerns | No concerns | No concerns | Some concerns | High |  |
| PAC100Bid:PAC400Qd | No concerns | Low risk | No concerns | No concerns | No concerns | Some concerns | High |  |
| PAC100Bid:PLB | No concerns | Low risk | No concerns | No concerns | No concerns | Some concerns | High |  |
| PAC100Bid:RUX | No concerns | Low risk | No concerns | No concerns | No concerns | Some concerns | High |  |
| PAC100Qd:PAC400Qd | No concerns | Low risk | No concerns | No concerns | No concerns | Some concerns | High |  |
| PAC100Qd:PLB | No concerns | Low risk | No concerns | No concerns | No concerns | Some concerns | High |  |
| PAC100Qd:RUX | No concerns | Low risk | No concerns | No concerns | No concerns | Some concerns | High |  |
| PAC200Bid:PLB | No concerns | Low risk | No concerns | No concerns | No concerns | Some concerns | High |  |
| PAC200Bid:RUX | No concerns | Low risk | No concerns | No concerns | No concerns | Some concerns | High |  |
| PAC400Qd:PLB | No concerns | Low risk | No concerns | No concerns | No concerns | Some concerns | High |  |
| PAC400Qd:RUX | No concerns | Low risk | No concerns | No concerns | No concerns | Some concerns | High |  |

**8.4 CINeMA confidence rating result of Anemia (grade 3or4)**

| **Comparison** | **Within-study bias** | **Reporting bias** | **Indirectness** | **Imprecision** | **Heterogeneity** | **Incoherence** | **Confidence rating** | **Reason(s) for downgrading** |
| --- | --- | --- | --- | --- | --- | --- | --- | --- |
| BAT:MMB200Qd | No concerns | Low risk | -- | No concerns | No concerns | No concerns | High |  |
| BAT:PAC200Bid | No concerns | Low risk | -- | No concerns | No concerns | No concerns | High |  |
| BAT:PAC400Qd | No concerns | Low risk | -- | No concerns | No concerns | No concerns | High |  |
| BAT:RUX | No concerns | Low risk | -- | No concerns | No concerns | No concerns | High |  |
| FED300Qd:FED400Qd | No concerns | Low risk | -- | No concerns | No concerns | No concerns | High |  |
| FED300Qd:FED500Qd | No concerns | Low risk | -- | No concerns | No concerns | No concerns | High |  |
| FED400Qd:FED500Qd | No concerns | Low risk | -- | No concerns | No concerns | No concerns | High |  |
| FED400Qd:PLB | No concerns | Low risk | -- | No concerns | No concerns | No concerns | High |  |
| FED500Qd:PLB | No concerns | Low risk | -- | No concerns | No concerns | No concerns | High |  |
| MMB200Qd:RUX | No concerns | Low risk | -- | No concerns | No concerns | No concerns | High |  |
| PAC100Bid:PAC100Qd | No concerns | Low risk | -- | No concerns | No concerns | No concerns | High |  |
| PAC100Bid:PAC200Bid | No concerns | Low risk | -- | No concerns | No concerns | No concerns | High |  |
| PAC100Qd:PAC200Bid | No concerns | Low risk | -- | No concerns | No concerns | No concerns | High |  |
| PAC200Bid:PAC400Qd | No concerns | Low risk | -- | No concerns | No concerns | No concerns | High |  |
| PLB:RUX | No concerns | Low risk | -- | No concerns | No concerns | No concerns | High |  |
| BAT:FED300Qd | No concerns | Low risk | -- | No concerns | Some concerns | Some concerns | High |  |
| BAT:FED400Qd | No concerns | Low risk | -- | No concerns | No concerns | Some concerns | High |  |
| BAT:FED500Qd | No concerns | Low risk | -- | No concerns | No concerns | Some concerns | High |  |
| BAT:PAC100Bid | No concerns | Low risk | -- | No concerns | No concerns | Some concerns | High |  |
| BAT:PAC100Qd | No concerns | Low risk | -- | No concerns | No concerns | Some concerns | High |  |
| BAT:PLB | No concerns | Low risk | -- | No concerns | No concerns | Some concerns | High |  |
| FED300Qd:MMB200Qd | No concerns | Low risk | -- | No concerns | Some concerns | Some concerns | High |  |
| FED300Qd:PAC100Bid | No concerns | Low risk | -- | No concerns | Some concerns | Some concerns | High |  |
| FED300Qd:PAC100Qd | No concerns | Low risk | -- | No concerns | Some concerns | Some concerns | High |  |
| FED300Qd:PAC200Bid | No concerns | Low risk | -- | No concerns | Some concerns | Some concerns | High |  |
| FED300Qd:PAC400Qd | No concerns | Low risk | -- | No concerns | Some concerns | Some concerns | High |  |
| FED300Qd:PLB | No concerns | Low risk | -- | No concerns | Some concerns | Some concerns | High |  |
| FED300Qd:RUX | No concerns | Low risk | -- | No concerns | No concerns | Some concerns | High |  |
| FED400Qd:MMB200Qd | No concerns | Low risk | -- | No concerns | No concerns | Some concerns | High |  |
| FED400Qd:PAC100Bid | No concerns | Low risk | -- | No concerns | No concerns | Some concerns | High |  |
| FED400Qd:PAC100Qd | No concerns | Low risk | -- | No concerns | No concerns | Some concerns | High |  |
| FED400Qd:PAC200Bid | No concerns | Low risk | -- | No concerns | No concerns | Some concerns | High |  |
| FED400Qd:PAC400Qd | No concerns | Low risk | -- | No concerns | No concerns | Some concerns | High |  |
| FED400Qd:RUX | No concerns | Low risk | -- | No concerns | No concerns | Some concerns | High |  |
| FED500Qd:MMB200Qd | No concerns | Low risk | -- | No concerns | No concerns | Some concerns | High |  |
| FED500Qd:PAC100Bid | No concerns | Low risk | -- | No concerns | No concerns | Some concerns | High |  |
| FED500Qd:PAC100Qd | No concerns | Low risk | -- | No concerns | No concerns | Some concerns | High |  |
| FED500Qd:PAC200Bid | No concerns | Low risk | -- | No concerns | No concerns | Some concerns | High |  |
| FED500Qd:PAC400Qd | No concerns | Low risk | -- | No concerns | No concerns | Some concerns | High |  |
| FED500Qd:RUX | No concerns | Low risk | -- | No concerns | No concerns | Some concerns | High |  |
| MMB200Qd:PAC100Bid | No concerns | Low risk | -- | No concerns | No concerns | Some concerns | High |  |
| MMB200Qd:PAC100Qd | No concerns | Low risk | -- | No concerns | No concerns | Some concerns | High |  |
| MMB200Qd:PAC200Bid | No concerns | Low risk | -- | No concerns | No concerns | Some concerns | High |  |
| MMB200Qd:PAC400Qd | No concerns | Low risk | -- | No concerns | No concerns | Some concerns | High |  |
| MMB200Qd:PLB | No concerns | Low risk | -- | No concerns | No concerns | Some concerns | High |  |
| PAC100Bid:PAC400Qd | No concerns | Low risk | -- | No concerns | No concerns | Some concerns | High |  |
| PAC100Bid:PLB | No concerns | Low risk | -- | No concerns | No concerns | Some concerns | High |  |
| PAC100Bid:RUX | No concerns | Low risk | -- | No concerns | No concerns | Some concerns | High |  |
| PAC100Qd:PAC400Qd | No concerns | Low risk | -- | No concerns | No concerns | Some concerns | High |  |
| PAC100Qd:PLB | No concerns | Low risk | -- | No concerns | No concerns | Some concerns | High |  |
| PAC100Qd:RUX | No concerns | Low risk | -- | No concerns | No concerns | Some concerns | High |  |
| PAC200Bid:PLB | No concerns | Low risk | -- | No concerns | No concerns | Some concerns | High |  |
| PAC200Bid:RUX | No concerns | Low risk | -- | No concerns | No concerns | Some concerns | High |  |
| PAC400Qd:PLB | No concerns | Low risk | -- | No concerns | No concerns | Some concerns | High |  |
| PAC400Qd:RUX | No concerns | Low risk | -- | No concerns | No concerns | Some concerns | High |  |
